# The *PtrC2H2.2‐6‐PtrCYP86A7/A8* Module Regulates Poplar Drought Tolerance Through Mediating Cutin and Wax Biosynthesis Pathways

**DOI:** 10.1111/pbi.70419

**Published:** 2025-10-24

**Authors:** Jiu‐Jiu Zhao, Shuang‐Lian Deng, Hao Li, Shu‐Ying Wei, Rui‐Quan Wang, Yuan Zhang, Xiang Xiang, Peng Yang, Jing Li, Ting Wang, Jinliang Huang, Liang‐Hua Chen, HanBo Yang, Xue‐Qin Wan, Fang He

**Affiliations:** ^1^ Forest Ecology and Conservation in the Upper Reaches of the Yangtze River Key Laboratory of Sichuan Province, Sichuan Mt. Emei Forest Ecosystem National Observation and Research Station, College of Forestry Sichuan Agricultural University Chengdu China

**Keywords:** drought, *Populus*, *PtrC2H2.2‐6*, *PtrCYP86A7*, *PtrCYP86A8*

## Abstract

The plant cuticle, composed of cutin and wax, is crucial for reducing non‐stomatal water loss and enhancing drought tolerance. *CYP86A* genes are key regulators of cutin and wax biosynthesis, yet the mechanisms controlling their expression remain unclear. Here, we identified *PtrC2H2.2‐6*, a C2H2‐type transcription factor that is downregulated at both transcriptional and protein levels under water stress in poplar. Through bioinformatics and biochemical analyses, we demonstrated that PtrC2H2.2‐6 interacts with PtrPPK1 to regulate drought tolerance by modulating two critical cuticle biosynthesis genes, *PtrCYP86A7* and *PtrCYP86A8*. *PtrC2H2.2‐6* negatively regulates cutin and wax biosynthesis by binding to the CACT motif in the promoters of *PtrCYP86A7* and *PtrCYP86A8*, thereby suppressing their expression. Conversely, PtrPPK1 interacts with PtrC2H2.2‐6, phosphorylates it, and promotes its degradation, thereby relieving its repression of *PtrCYP86A7/8*. Functional validation revealed that RNAi‐mediated silencing of *PtrC2H2.2‐6* enhanced wax accumulation and drought tolerance, while overexpression of *PtrC2H2.2‐6* reduced wax accumulation and drought resistance. Furthermore, overexpression of *PtrCYP86A7* or *PtrCYP86A8* increased wax accumulation, enhanced the water retention capacity of the leaf surface, and improved drought resistance. Collectively, the *PtrPPK1*‐*PtrC2H2.2‐6*‐*PtrCYP86A7/A8* module is a critical regulatory mechanism for drought tolerance in poplar, offering potential targets for breeding drought‐resistant forest trees.

## 1 Introduction

In response to escalating global drought conditions, plants have developed intricate molecular mechanisms to mitigate water scarcity. Key adaptations include regulating stomatal closure (Yang et al. 2022), scavenging reactive oxygen species (Yu et al. 2024) and enhancing the biosynthesis of cuticle‐related substances (Seo et al. 2011). These mechanisms are essential for plant resilience, ensuring survival and productivity under environmental stress. Among these, the cuticle, which makes up the outermost layer of protection for plants, is essential for protecting them from biotic and abiotic threats. It prevents non‐stomatal water loss (Zhang et al. 2007; Kosma et al. 2009), protects against ultraviolet radiation damage (Arya et al. 2021), and defends against pests and pathogens (Arya et al. 2021).

The cuticle comprises two primary components: cutin and wax. Cutin forms the structural backbone of the cuticle, synthesised through polymerisation of ω‐hydroxy fatty acids with 16‐ and 18‐carbon chains and the cross‐linking of secondary hydroxyl groups (Nawrath 2006; Heredia 2003). Wax, a complex mixture of very long‐chain fatty acids (VLCFAs) and their derivatives, includes alkanes, ketones, aldehydes, alcohols and esters (Wang et al. 2017). Together, these components create a robust barrier critical for plant adaptation to stress and overall survival (Bhanot et al. 2021). The synthesis of the cuticle is influenced by environmental and intrinsic factors. External factors such as light intensity and quality (Lee and Suh 2022; Xu et al. 2024) and temperature fluctuations (Shepherd and Wynne Griffiths 2006; Shaheenuzzamn et al. 2021) significantly regulate cuticle development.

The cytochrome P450 (CYP450) family, a class of multifunctional monooxygenases, is involved in cuticle synthesis by catalysing a series of key oxidative reactions (Zhang et al. 2020). For instance, *CYP77A6* is involved in the oxidation of medium‐chain fatty acids (Li‐Beisson et al. 2009), while *CYP96A15* oxidises alkanes to produce secondary alcohols and ketones (Greer et al. 2007). Additionally, members of the CYP86A family catalyse the ω‐hydroxylation of fatty acids ranging from C12 to C18 (Xiao et al. 2004; Lee and Suh 2013). In addition to P450 enzymes, other genes are implicated in cuticle biosynthesis. For example, *KCS2* and *KCS11* in Citrus enhance leaf wax content (Yang, Mei, et al. 2021), while *CER1* influences wax density in maize, with mutants reducing wax and overexpression increasing it (Zhao, Liu, et al. 2024). Transcription factors also play a pivotal role. Overexpression of *MdSHINE2* in apples alters epidermal wax morphology and enhances drought resistance (Zhang et al. 2019). In rice, *OsMYB60* directly upregulates *OsCER1*, boosting leaf wax synthesis and drought tolerance (Jian et al. 2022). The transcription factor *WIN1* similarly regulates cuticle biosynthesis by activating genes encoding critical biosynthetic enzymes (Kannangara et al. 2007). In summary, cuticle biosynthesis is a complex process involving the synergistic action of multiple genes.

Although previous studies have identified genes in poplar involved in cuticle wax and cutin biosynthesis, such as *PtoMYB142*, which controls wax synthesis by directly binding to the promoter regions of *CER4* and *KCS6* in *Populus trichocarpa* (Song et al. 2022), research on the role of P450 genes in poplar cuticle biosynthesis remains limited. In particular, the regulation of these genes by upstream transcription factors is poorly understood. In our prior work, we characterised the C2H2 transcription factor family and identified *PtrC2H2.2‐6*, also referred to as *PtrDSC1* (Drought‐Sensitive C2H2‐like Transcription Factor 1 in Poplar), as a critical member. It was discovered that under osmotic stress, this factor was negatively controlled (Zhao, Xiang, et al. 2024). Bioinformatics analysis suggested that *PtrDSC1* might regulate *PtrCYP86A7* (the poplar most homologous gene of Arabidopsis *CYP86A7*) and *PtrCYP86A8* (the poplar most homologous gene of Arabidopsis *CYP86A8*), but the precise physiological and molecular processes by which the *PtrDSC1*‐*PtrCYP86A7*/*A8* module modulates cuticle and wax biosynthesis—and thereby drought tolerance—are yet to be elucidated.

This research aims to fill this knowledge gap by investigating the role of *PtrC2H2.2‐6* in the drought stress response and its potential regulation of *PtrCYP86A7* and *PtrCYP86A8*. Using a combination of plant physiology, molecular biology and bioinformatics approaches, we will analyse the mechanisms through which the *PtrC2H2.2‐6*‐*PtrCYP86A7*/*A8* module governs poplar's response to drought stress. These results will offer essential insights into the molecular foundations of drought resistance in poplar trees. By elucidating the impact of the cuticle on drought resistance and unravelling the hierarchical regulatory network underlying poplar's drought response, this research fills a significant knowledge gap. In addition, the results will offer valuable targets and theoretical foundations for the genetic design and development of drought‐resistant forest tree species. This work not only advances our understanding of poplar's molecular response to drought but also paves the way for innovative molecular breeding strategies to enhance adaptability to future climate challenges.

## 2 Materials and Methods

### 2.1 Construction of Vectors and Genetic Transformation of Poplar

Using homologous recombination technology, all transgenic vectors were successfully created, including recombinant vectors with the *PtrC2H2.2‐6/PtrCYP86A7/PtrCYP86A8* genes driven by the 35S promoter and an RNA interference (RNAi) vector that expressed the *PtrC2H2.2‐6* gene using the pCAMBIA2301 vector (He et al. 2019). The 84 K Poplar ( *Populus alba* × *Populus glandulosa*) genetic transformation technique was based on a prior paper (He et al. 2018). RNAi‐*PtrC2H2.2‐6* and Ox‐*PtrC2H2.2‐6* transgenic poplars were derived from previous studies (Zhao, Liu, et al. 2024).

### 2.2 Scanning Electron Microscope (SEM) Shooting

Through scanning electron microscopy, mature leaves from various transgenic lines and the wild type that were the same age and location were chosen. Refer to the instructions provided in Methods S10 for detailed procedures on leaf preparation. A scanning electron microscope (ZEISS Sigma 300, Germany) was then used to view the prepared leaves' upper surface and photographed at a magnification of 20 000×, with each line containing at least three biological replicates.

### 2.3 Fourier Transform Infrared Spectral (FTIR) Analysis

Mature leaves from the same parts of 4‐week‐old transgenic and wild‐type plants were selected and freeze‐dried in a vacuum for 24 h. About 2 mg of sample was weighed and ground together with 200 mg of dried potassium bromide and finally placed in a moulder and vacuumed to make sample flakes, which were analysed by Fourier transform infrared spectroscopy (FTIR) using an infrared spectrometer—INVENIOS (Bruker, Germany) (Khan et al. 2018). There were at least three biological replicates for each treatment. The collected data were analysed in detail using Origin software and characterised the functional groups of cutin and wax‐related metabolites based on wavelength characteristics.

### 2.4 Wax Extraction and GC–MS Analysis

For GC–MS analysis, leaves were taken from the same parts of transgenic and wild‐type plants that were 4 weeks old. The extraction of wax and subsequent analysis by GC–MS was conducted by Nanjing Ruiyuan Biotechnology Co. Ltd. For detailed procedures, please refer to Methods S12.

### 2.5 Transcriptional Regulatory Network and Protein Interaction Network of the PtrC2H2.2‐6

Differentially expressed genes (DEGs) identified in the transcriptome data were combined and analysed with potential downstream target genes of *PtrC2H2.2‐6* predicted by the PlantTFDB database (Tian et al. 2020) to identify target genes. Subsequently, we performed functional annotation analysis of these target genes using the TAIR database. The co‐expression heatmaps of these target genes in wild type and *PtrC2H2.2‐6* overexpression plants were constructed by TBtools v2.0 (Chen et al. 2020). Meanwhile, we also mapped the functional regulatory network between *PtrC2H2.2‐6* and its target genes using Cytoscape software (Shannon et al. 2003). Regarding the protein interaction network, specifically, protein screen library experiments were performed to initially identify proteins that may interact with *PtrC2H2.2‐6*; all were genetically annotated and subsequently mapped using Cytoscape software.

### 2.6 DNA Affinity Purification PCR (DAP‐PCR)

Total plant DNA was extracted from *P. trichocarpa* , resulting in DNA fragments sized between 100 and 500 bp through the use of ultrasonic fragmentation. The purification of GST fusion proteins was performed using BeyoGold GST‐tag Purification Resin (Beyotime, P2251, Beijing, China). The DNA fragments were co‐incubated with GST‐*PtrC2H2.2‐6*‐HA for 4 h in an incubation buffer that included 50 mM Tris, 1 mM EDTA and 100 mM KCl, with the pH adjusted to 7.0 using hydrochloric acid, in addition to 5% glycerol and 0.1% Triton X‐100. To achieve a final concentration of 1 mM, freshly prepared 100 mM DTT was incorporated into the reaction mixture. After co‐incubation, the glutathione‐agarose beads underwent three washes with the incubation buffer. Following this, 4 μL of a 5 M NaCl solution was added for every 100 μL of the sample and allowed to incubate for 4 h to facilitate the dissociation of the cross‐linked GST‐*PtrC2H2.2‐6*‐HA from the DNA fragments (Li et al. 2017). DNA fragments were then extracted using the phenol‐chloroform method, and the eluted DNA fragments were used in downstream PCR experiments to assess the possibility of interactions between the PtrC2H2.2‐6 proteins and downstream promoters of related genes. For specific steps to operate DAP‐PCR, please refer to the relevant published literature (Lin et al. 2013).

### 2.7 Electrophoretic Mobility Shift Assays

The most likely binding motifs for *PtrC2H2.2‐6* and *PtrCYP86A7/A8* were identified based on the predicted target gene binding sites in DAP‐PCR and the Plant Transcription Factor Database (Plant Reg Map/PlantTFDB v5.0), respectively. The wild probe was the putative *PtrC2H2.2‐6* TFBD (CTCTCTCACT) on the *PtrCYP86A7* promoter, and the putative TFBD sequence CTCTCTCACT in the mutant probe was replaced by CCCTCTCCCT. The wild probe was the putative *PtrC2H2.2‐6* TFBD (CACCCTCACT) on the *PtrCYP86A8* promoter, and the putative TFBD sequence CACCCTCACT was replaced by CCCCCTCCCT in the mutant probe. Sangon (Beijing, China) manufactured the binding motif fragments. The following EMSA tests were conducted in accordance with the manufacturer's instructions using the EMSA Probe Biotin Labelling kit and a chemiluminescent EMSA kit (GS008 and GS009; Beyotime Biotechnology, Shanghai, China).

### 2.8 In Vitro Pull‐Down Assay

We cloned the full‐length coding sequences (CDS) of *PtrC2H2.2‐6* and *PtrPPK1* into the PGEX‐5X‐1 and PET32A vectors, respectively. The recombinant vectors, GST‐*PtrC2H2.2‐6*‐HA and *PtrPPK1*‐His, were then used to express the target proteins in *Escherichia coli* BL21. The protein was then induced using 0.5 mM isopropyl β‐D‐1‐thiogalactopyranoside (IPTG). In compliance with the manufacturer's instructions, his fusion proteins and GST fusion proteins were purified using the Ni NTA Beads 6FF gravity columns (Smart‐Lifesciences, SA005GC01, Changzhou, China) and the Glutathione Beads 4FF gravity columns (Smart‐Lifesciences, SA010GC01, Changzhou, China), respectively. Soluble GST‐HA or GST‐*PtrC2H2.2‐6*‐HA fusion proteins were extracted and fixed with BeyoGold GST‐tag Purification Resin (Beyotime, P2251, Beijing, China). PtrPPK1‐His proteins were incubated with immobilised GST‐HA or GST‐PtrC2H2.2‐6‐HA proteins, and their interaction was detected by Western blot analysis using anti‐GST (Beyotime, AF0174, Beijing, China) and anti‐His antibodies (Sigma‐Aldrich). Table S5 contains a list of primers.

### 2.9 In Vitro Protein Degradation Assay

Mature leaves of poplar trees of the same site at 4 weeks of age were collected and treated with pure water, 30% PEG, and 100 μM ABA for 12 h. Total poplar protein was extracted using RIPA containing 1 mM PMSF, and protein loading buffer (6×) was added for subsequent up‐sampling. Protein extracts were incubated with GST‐PtrC2H2.2‐6‐HA fusion proteins and collected at room temperature for different time periods (0, 1, 2 and 3 h). Western blotting using an anti‐GST antibody (Beyotime, AF0174, Beijing, China) was used to detect the samples. As previously mentioned, the GST‐PtrC2H2.2‐6‐HA protein signal intensities were measured independently using ImageJ (Wu et al. 2016).

### 2.10 In Vivo Protein Degradation Assay

Using Agrobacterium‐mediated transient transformation, the same concentrations of 35S:*PtrC2H2.2‐6* and 35S:*PtrPPK1* or 35S empty vector bacteriophage were transferred into tobacco (*Nicotiana benthamiana*) leaves of the same condition. After co‐transformation of tobacco, leaves were harvested after 1 day of darkness and 1 day of light, and a 50 μM MG132 treatment was applied to the leaves after 12 h of light leaf exposure. Immunoblotting assays using an anti‐GFP antibody (Beyotime, AF0159, Beijing, China) were used to detect PtrC2H2.2‐6‐GFP protein levels. As previously mentioned, the signal intensity of PtrC2H2.2‐6‐GFP proteins was measured independently using ImageJ (Wu et al. 2016).

### 2.11 Statistical Analysis

SPSS and Microsoft Excel 2020 were used for the data analysis. To determine the significance of the variations among the treatments, a one‐way ANOVA was conducted. *p* values were determined using a student's *t*‐test (*, *p* ≤ 0.05; **, *p* ≤ 0.01).

## 3 Results

### 3.1 Thorough Molecular Analysis and Unique Expression Patterns of *PtrC2H2.2‐6*

**FIGURE 1:**
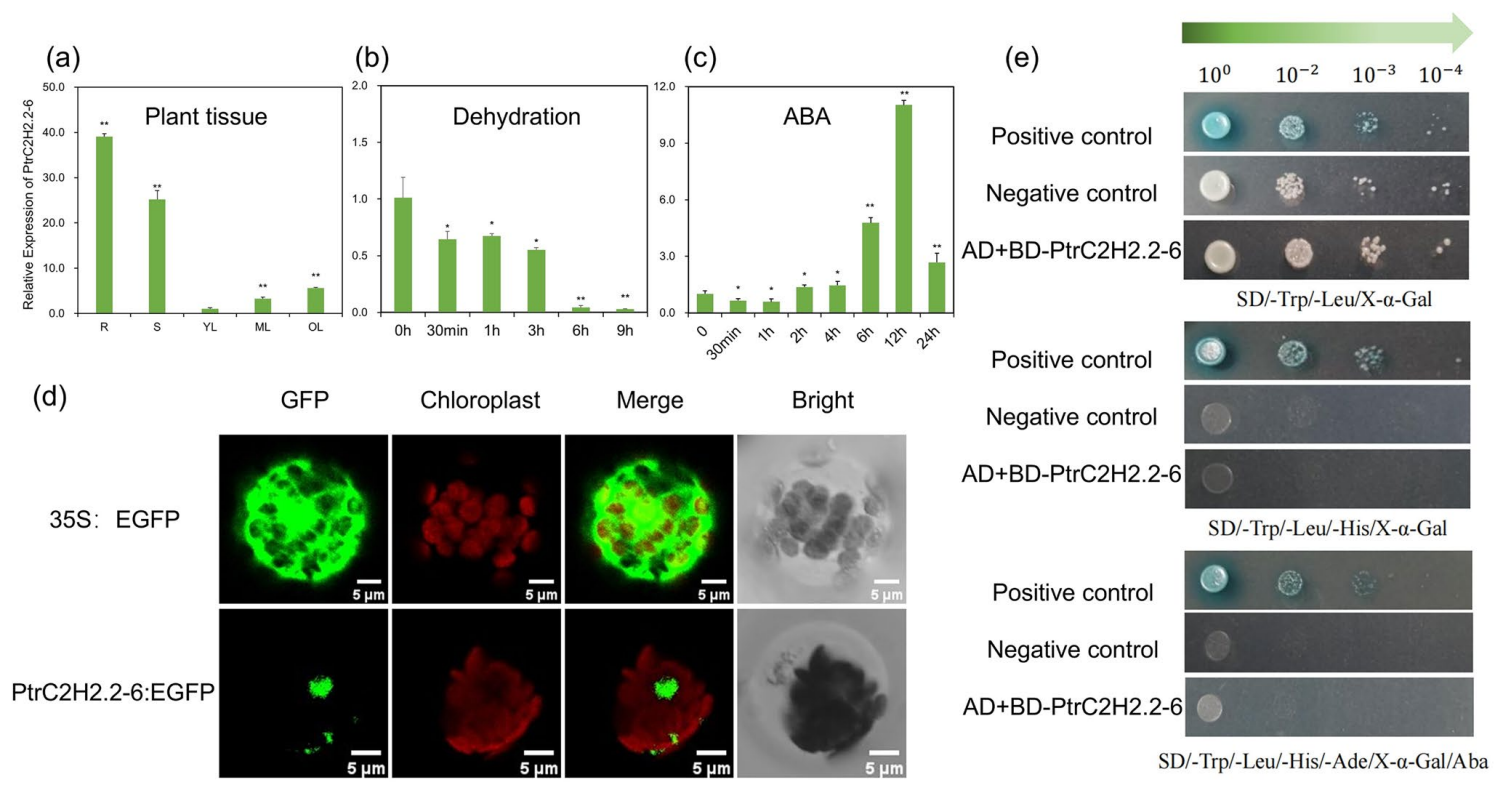
Molecular characterisation of *PtrC2H2.2‐6*. (a) The transcript levels of *PtrC2H2.2‐6* in different tissues of *P. trichocarpa* . R, root; S, stem; YL, young leaf; ML, mature leaf; OL, old leaf. (b) The transcript levels of *PtrC2H2.2‐6* in leaf tissues were monitored at different times of dehydration. (c) The transcript levels of *PtrC2H2.2‐6* in leaf tissues were evaluated at various time points following ABA treatment. Values are means ± SD (*n* = 9). Asterisks denote significant differences. *, *p* ≤ 0.05; **, *p* ≤ 0.01. (d) Subcellular localization of 35S: GFP and 35S: *PtrC2H2.2‐6*‐GFP in transiently expressed Arabidopsis leaf protoplasts. Red fluorescent protein (RFP) is used to indicate chloroplasts. Bars = 5 μm. (e) Validation of self‐activation of *PtrC2H2.2‐6*. BK‐*PtrC2H2.2‐6* + AD null cultured on SD/−Trp/Leu, SD/−Trp/Leu/‐His and SD/−Trp/Leu/‐His/−Ade. pGBKT7‐53 and pGADT7‐T as positive controls, pGBKT7‐Lam + pGADT7‐T as a negative control.

To investigate the potential function of *PtrC2H2.2‐6*, we analysed its expression levels across various tissues of *P. trichocarpa* . The gene showed high expression in mature leaves, old leaves, roots and stems (Figure 1a; Methods S1, S2). Further examination revealed that its expression gradually declined during prolonged leaf dehydration (Figure 1b; Methods S1, S2). Additionally, we evaluated *PtrC2H2.2‐6* expression following abscisic acid (ABA) treatment. The expression exhibited dynamic fluctuations. Initially, it decreased, reaching its nadir at 1 h. Subsequently, the expression levels began to climb, culminating in a peak at the 12‐h mark (Figure 1c; Methods S1, S2). To ascertain the subcellular localization, we combined *PtrC2H2.2‐6* with green fluorescent protein (GFP) regulated by the 35S promoter and transiently introduced this construct into Arabidopsis protoplasts. The GFP signal indicated that *PtrC2H2.2‐6* is localised in the nucleus (Figure 1d; Methods S3). Furthermore, a yeast self‐activation assay was conducted to evaluate its transcriptional activation activity. The BD‐*PtrC2H2.2‐6* fusion protein failed to grow on triple‐deficient (S/−T/−L/−H) and quadruple‐deficient (S/−T/−L/−H/−A) medium, confirming that *PtrC2H2.2‐6* lacks self‐activating activity (Figure 1e; Methods S4).

### 3.2 *PtrC2H2.2‐6* Diminishes Poplar's Capacity to Withstand Drought

**FIGURE 2:**
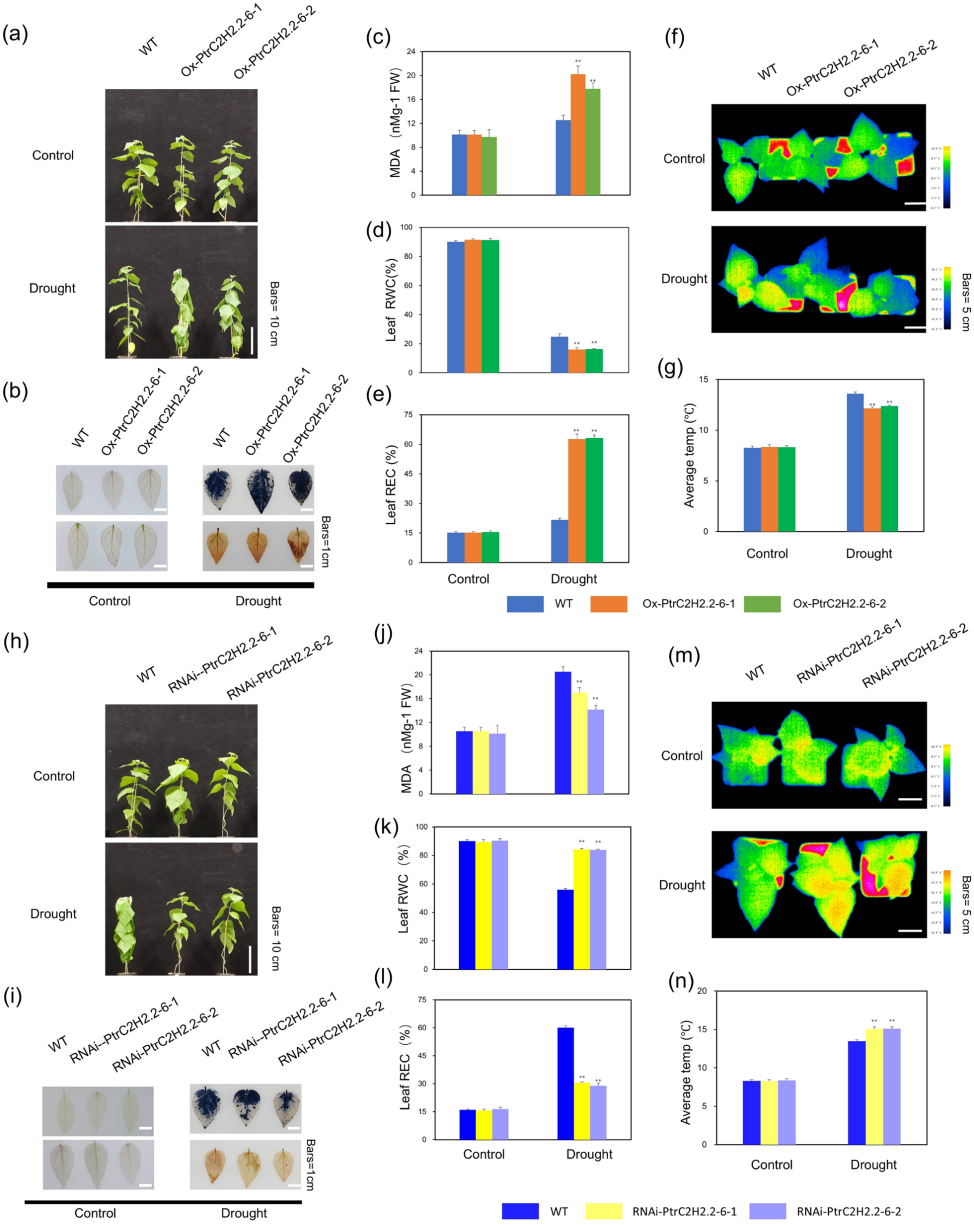
Phenotypic analysis of *PtrC2H2.2‐6* regulation of poplar response to drought. (a) Morphological differences between Ox‐*PtrC2H2.2‐6* and the wild type in short‐term drought assays. Bars = 10 cm. (b) DAB and NBT staining for the detection of O_2_^−^ and H_2_O_2_ in leaves of Ox‐*PtrC2H2.2‐6* and wild‐type plants after short‐term drought treatment. Bars = 1 cm. MDA content (c), quantitative measurement of RWC (relative water content) (d) and quantitative measurement of REC (relative electrical conductance) (e) in the leaves of wild‐type and Ox‐*PtrC2H2.2‐6* plants under normal and short drought stress conditions. Values are means ± SD (*n* = 9). Asterisks denote significant differences. *, *p* ≤ 0.05; **, *p* ≤ 0.01. (f) Infrared thermography of leaf temperature in Ox‐*PtrC2H2.2‐6* and wild‐type poplar after drought treatment. Colour bars represent different temperatures. (g) Statistical analysis of leaf temperatures in (f). Values are means ± SD (*n* = 9). Asterisks denote significant differences. *, *p* ≤ 0.05; **, *p* ≤ 0.01. (h) Morphological differences between RNAi‐*PtrC2H2.2‐6* and the wild type in short‐term drought assays. Bars = 10 cm. (i) DAB and NBT staining for the detection of O_2_^−^ and H_2_O_2_ in RNAi‐*PtrC2H2.2‐6* and wild‐type plants after short‐term drought treatment. Bars = 1 cm. MDA content (j), quantitative measurement of RWC (relative water content) (k), and quantitative measurement of REC (relative electrical conductance) (l) in the leaves of wild‐type and RNAi‐*PtrC2H2.2‐6* plants under normal and short drought stress conditions. Values are means ± SD (*n* = 9). Asterisks denote significant differences. *, *p* ≤ 0.05; **, *p* ≤ 0.01. (m) Infrared thermography of leaf temperature in Ox‐*PtrC2H2.2‐6* and wild‐type poplar after drought treatment. Colour bars represent different temperatures. (n) Statistical analysis of leaf temperatures in (m). Values are means ± SD (*n* = 9). Asterisks denote significant differences. *, *p* ≤ 0.05; **, *p* ≤ 0.01.

To clarify the functional role of *PtrC2H2.2‐6*, we conducted drought stress experiments using three genotypes of poplar: overexpressing, wild type and RNA‐interfering. While RNAi‐*PtrC2H2.2‐6* poplars maintained superior growth conditions after drought treatment, Ox‐*PtrC2H2.2‐6* poplars showed notable wilting in comparison to the wild type (Figure 2a,h; Methods S5). Ox‐*PtrC2H2.2‐6* poplars accumulated more O_2_^−^ and H_2_O_2_ than the wild type, but RNAi‐*PtrC2H2.2‐6* poplars showed less accumulation, according to DAB and NBT staining (Figure 2b,i; Methods S7). The relative water content (RWC), a critical measure of leaf hydration under stress, decreased significantly in all genotypes under short‐term drought treatment. However, the reduction was most pronounced in Ox‐*PtrC2H2.2‐6* plants and least severe in RNAi‐*PtrC2H2.2‐6* plants (Figure 2d,k; Methods S6). Malondialdehyde (MDA) levels, an indicator of cell membrane damage, increased in all genotypes under drought stress. Ox‐*PtrC2H2.2‐6* plants exhibited the highest MDA accumulation, while RNAi‐*PtrC2H2.2‐6* plants showed comparatively lower levels (Figure 2c,j; Methods S6). Consistently, relative electrical conductivity (REC), which reflects membrane integrity, rose significantly across all genotypes following drought treatment. The Ox‐*PtrC2H2.2‐6* plants exhibited the highest REC, indicating severe membrane damage, whereas the RNAi‐*PtrC2H2.2‐6* plants had the lowest REC, signifying enhanced membrane stability (Figure 2e,l; Methods S6). Thermal imaging analysis further supported these findings. Infrared thermography showed that Ox‐*PtrC2H2.2‐6* plants had lower leaf temperatures post‐drought, indicative of increased water loss, while RNAi‐*PtrC2H2.2‐6* plants maintained higher leaf temperatures, reflecting reduced transpiration (Figure 2f,g,m,n; Methods S8). Overall, these results demonstrate that *PtrC2H2.2‐6* adversely controls resilience to drought in poplars. Silencing this gene improved drought resistance by mitigating water loss, minimising oxidative harm and enhancing membrane integrity.

### 3.3 *PtrC2H2.2‐6* Affects electron Transport Rate in Plant Photosynthetic System II Under Drought Stress

**FIGURE 3:**
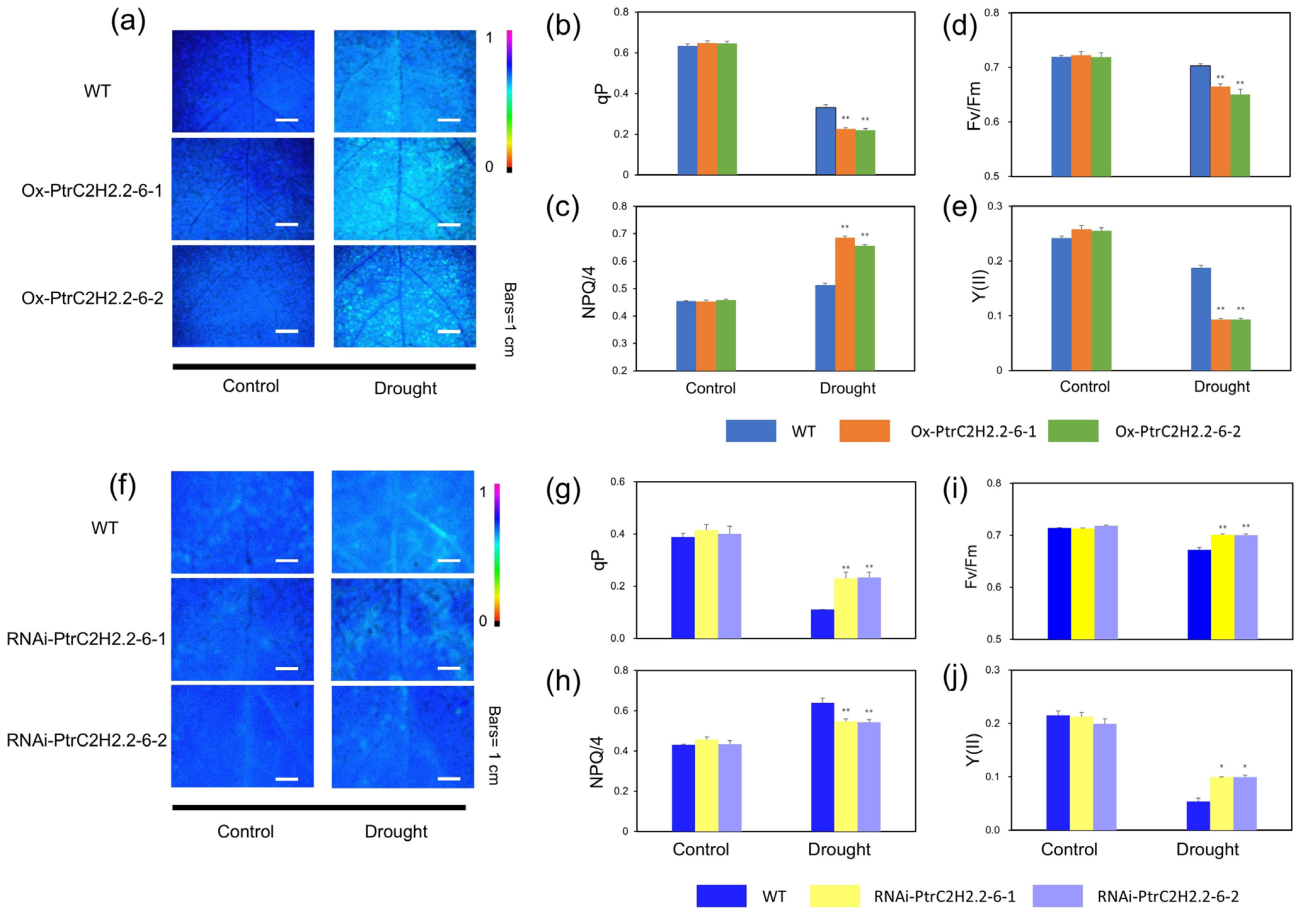
The parameters of the slow dynamic fluorescence induction curve were measured in Ox‐*PtrC2H2.2‐6*, RNAi‐*PtrC2H2.2‐6* and WT plants under normal and short drought stress conditions. (a) Maximum potential photochemical efficiency (*F*_v_/*F*_m_) fluorescence plot, photochemical quenching parameter (qP) (b), non‐photochemical quenching parameter/4 (NPQ/4) (c), maximal PSII quantum yield (*F*_v_/*F*_m_) (d), quantum yield of photochemical energy conversion in PSII (Y (II)) (e) in the leaves of wild type and Ox‐*PtrC2H2.2‐6* plants under normal and short drought stress conditions. (f) Maximum potential photochemical efficiency (*F*_v_/*F*_m_) fluorescence plot, photochemical quenching parameter (qP) (g), non‐photochemical quenching parameter/4 (NPQ/4) (h), maximal PSII quantum yield (*F*_v_/*F*_m_) (i), quantum yield of photochemical energy conversion in PSII (Y (II)) (j) in the leaves of wild type and RNAi‐*PtrC2H2.2‐6* plants under normal and short drought stress conditions. Values are means ± SD (*n* = 9). Asterisks denote significant differences. *, *p* ≤ 0.05; **, *p* ≤ 0.01.

To investigate whether *PtrC2H2.2‐6* influences photosynthesis, we measured chlorophyll fluorescence parameters in different poplar genotypes under drought stress. Drought impaired photosynthetic activity across all genotypes, as evidenced by reductions in photochemical quenching (qP), maximum photochemical efficiency (*F*_v_/*F*_m_), and quantum yield of photosystem II (Y(II)). However, the reductions were more pronounced in Ox‐*PtrC2H2.2‐6* poplars than in the wild type (Figure 3a,b,d,e; Methods S9), indicating that overexpression of *PtrC2H2.2‐6* exacerbates photosystem II's electron transportation deficiency. This suggests that wild‐type plants retain higher light energy conversion efficiency and photosynthetic activity under drought stress. Non‐photochemical quenching (NPQ/4), an indicator of photoprotective capacity, increased across all genotypes following drought treatment. However, Ox‐*PtrC2H2.2‐6* plants exhibited higher NPQ/4 levels compared to the wild type, reflecting their higher dissipation of excess light energy as heat (Figure 3c).

Conversely, as shown in Figure 3f,g,i,j, qP, *F*_v_/*F*_m_ and Y(II) decreased in all plants after short‐term drought treatments, but the wild type exhibited more reductions compared to the RNAi‐*PtrC2H2.2‐6*, suggesting that the silenced lines demonstrated enhanced light energy conversion efficiency and photosynthetic activity. In addition, Figure 3h shows that NPQ/4 was elevated in all plants after drought, but it was more elevated in the wild type compared to RNAi‐*PtrC2H2.2‐6*. These results suggest that *PtrC2H2.2‐6* negatively impacts photosynthetic efficiency under drought stress by impairing electron transport in photosystem II. Silencing *PtrC2H2.2‐6* helps maintain photosynthetic capacity, thereby improving drought tolerance in poplar. Additionally, experimental data under sustained drought conditions further support this conclusion. Under 80% soil relative water content (SRWC), no significant differences were observed in the growth performance among the different lines. However, under 30% SRWC, the RNAi‐*PtrC2H2.2‐6* poplar exhibited significantly higher plant height, ground diameter and biomass of roots, stems, and leaves compared to the wild type, while the Ox‐*PtrC2H2.2‐6* poplar showed significantly lower values in these parameters than the wild type (Figure S11a–f).

### 3.4 *PtrC2H2.2‐6* Affects Cutin, Suberin and Wax Biosynthesis Pathways at the Transcriptional and Metabolic Levels

**FIGURE 4:**
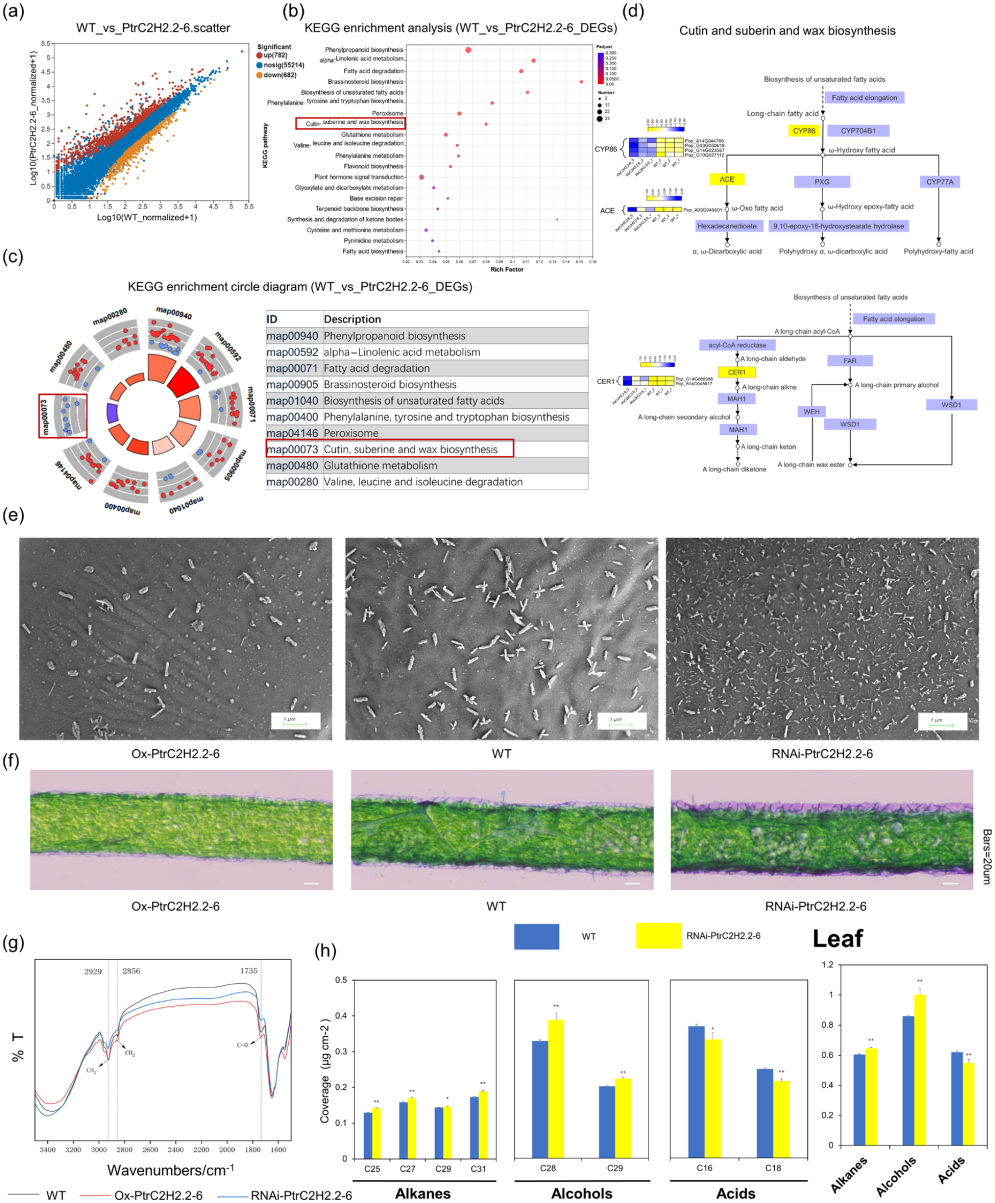
*PtrC2H2.2‐6* attenuates drought tolerance by affecting leaf surface cutin and wax biosynthesis in poplar trees. (a) The scatter diagram indicates differentially expressed genes (DEGs) in the Ox‐*PtrC2H2.2‐6* compared with wild‐type poplars. WT is the control group and Ox‐*PtrC2H2.2‐6* is the experimental group. The values of the horizontal and vertical coordinates were algorithmised, with the value of the horizontal coordinate corresponding to a particular point being the expression of the gene in the control sample and the value of the vertical coordinate being the expression of the gene in the treatment sample. Each dot represents a single gene. Red dots indicate significantly upregulated genes, orange dots indicate significantly down‐regulated genes, and blue points are non‐significantly different genes. (b) KEGG enrichment analysis of DEGs. The vertical axis represents the name of the pathway, the horizontal axis represents the Rich factor, the size of the dots indicates the number of genes in the pathway, and the colour of the dots corresponds to the different *p*‐adjust ranges. (c) KEGG enrichment circle diagram of DEGs. The upregulated and downregulated genes in the graph are coloured in red and blue, respectively, where the inner circle changes from purple to red for the *z*‐score value, which is used to estimate whether a biological process is likely to act. ID is shown in the figure, and the second column is the corresponding description information. (d) Schematic diagram of the synthesis process of cutin, suberin and wax biosynthesis and a heat map of the pathways of the DEGs therein. The yellow and blue bars (FPKM) indicate high and low expression, respectively. (e) SEM images of cuticular wax crystals on mature poplar leaves at the same position of 8‐week‐old WT and RNAi‐*PtrC2H2.2‐6* plants. Scale bars = 1 μm. (f) Microscope images of 6‐week‐old poplar leaves of different lines in the longitudinal section. Bars = 20 μm. (g) Fourier transform infrared spectroscopy (FTIR) of substances extracted from 8‐week‐old poplar same‐part mature leaves in different lines. The black line represents WT, the blue line represents Ox‐*PtrC2H2.2‐*6 and the red line represents RNAi‐*PtrC2H2.2‐6*. (h) Cuticular wax composition in the same‐part mature leaves of 4‐week‐old WT, Ox‐*PtrC2H2.2‐6* and RNAi‐*PtrC2H2.2‐6* plants. Values are means ± SD (*n* = 3). Asterisks denote significant differences. *, *p* ≤ 0.05; **, *p* ≤ 0.01.

To understand the intrinsic mechanisms underlying their response to drought, we performed RNA sequencing (RNA‐seq) on Ox*‐PtrC2H2.2‐6* and WT poplar to further elucidate the function of *PtrC2H2.2‐6* in drought response (Methods S13). 1464 DEGs, comprising 782 upregulated and 682 downregulated genes, were found in overexpressing plants (Figure 4a; Table S1). According to the KEGG enrichment analysis, these DEGs are associated with pathways such as phenylpropanoid biosynthesis, fatty acid metabolism and plant hormone signal transduction, especially with the biosynthesis pathway of cutin, suberin and wax (map00073) (Figure 4b; Table S7). In Ox‐*PtrC2H2.2‐6* poplar trees, only the biosynthesis pathway of cutin, suberin and wax (map00073) was significantly downregulated (Figure 4c). A heat map of DEGs involved in cutin, suberin and wax biosynthesis showed that key genes such as *CYP86A7*, *CYP86A8*, *CYP86C1*, *CER1* and *EDA17* showed lower expression in plants that were overexpressing as opposed to the wild type (Figure 4d; Table S4). Meanwhile, the co‐expression network showed that *PtrC2H2.2‐6* may combine with other transcription factors (MYB, NAC, bZIP, etc.) to synergistically regulate the expression of some of the above genes (Figure S12). Besides, among the DEGs, we also found some genes related to cutin and wax synthesis showing reduced expression in overexpressed plants, such as *KCS2*, *CER3* and *KCS11* (Figure S8; Table S1).

To further investigate this hypothesis, we employed scanning electron microscopy (SEM) to view the top layer of leaves of overexpressed, silenced and wild‐type plants. The SEM analysis revealed that RNAi‐*PtrC2H2.2‐6* leaf blades exhibited a greater formation of wax on their surfaces compared to the wild type, while Ox‐*PtrC2H2.2‐6* displayed a decreased presence of wax (Figure 4e). Meanwhile, staining of longitudinal sections of leaves of different lines revealed that the staining of longitudinal sections of leaves of RNAi‐*PtrC2H2.2‐6* plants was darker compared with that of the wild type, which implied that the content of cuticle‐related substances might be more abundant, whereas the staining of cuticle in longitudinal sections of leaves of Ox‐*PtrC2H2.2‐6* plants was relatively lighter, suggesting that the content of cuticle‐related substances might be relatively less (Figure 4f). Furthermore, the intensity of the characteristic functional groups of cutin and wax synthesis‐related substances absorption peaks varied significantly between the RNAi‐*PtrC2H2.2‐6* line, the Ox‐*PtrC2H2.2‐6* line and the wild type, according to the Fourier transform infrared spectroscopy (FTIR) study. Specifically, with respect to the wild type, the RNAi‐*PtrC2H2.2‐6* line had higher intensities at 2856 cm^−1^ (symmetric CH_2_ telescoping vibration), 2929 cm^−1^ (asymmetric CH_2_ telescoping vibration) and 1735 cm^−1^ (C=O telescoping vibration) (Figure 4g), which represent the characteristic functional groups of cutin and wax synthesis‐related substances (Arya et al. 2021). Conversely, the Ox‐*PtrC2H2.2‐6* line exhibited reduced absorption peak intensities at identical locations compared to the wild type, aside from at 2929 cm^−1^, where the intensity remained substantially the same. Moreover, wax crystals on the surface of RNAi‐*PtrC2H2.2‐6* plants were significantly more abundant than those on the wild type, which prompted us to analyse the core metabolites of the wax in the leaves of wild type and silenced plants using gas chromatography–mass spectrometry (GC–MS) (Figure S1b; Table S8). The principal component analysis (PCA) of GC–MS data showed significant differences in wax‐related metabolite contents between wild‐type poplar trees and RNAi‐*PtrC2H2.2‐6* poplar trees. The first principal component (PC1) accounted for 83.8% of the variation in the samples, confirming the reliability of the GC–MS results (Figure S1a). As shown in Figure 4h, in the leaves of RNAi‐*PtrC2H2.2‐6*, we observed higher concentrations of alkanes (C25, C27, C29 and C31) and primary alcohols (C28 and C29) involved in wax synthesis than in the wild type. However, the concentration of C28 and C29 fatty acids in the leaves of this plant was reduced compared to the wild type. Overall, the contents of alkanes and alcohols in the leaf wax of RNAi‐*PtrC2H2.2‐6* were significantly higher than those in wild‐type poplar trees. In summary, the results above imply that *PtrC2H2.2‐6* affects the cutin, suberin and wax biosynthesis pathway (map00073) at the transcriptional and metabolic levels.

### 3.5 *PtrC2H2.2‐6* Directly Regulates and Suppresses the Expression of Important Genes (*PtrCYP86A7/A8*) Involved in the Cutin, Suberin and Wax Biosynthesis Pathway

**FIGURE 5:**
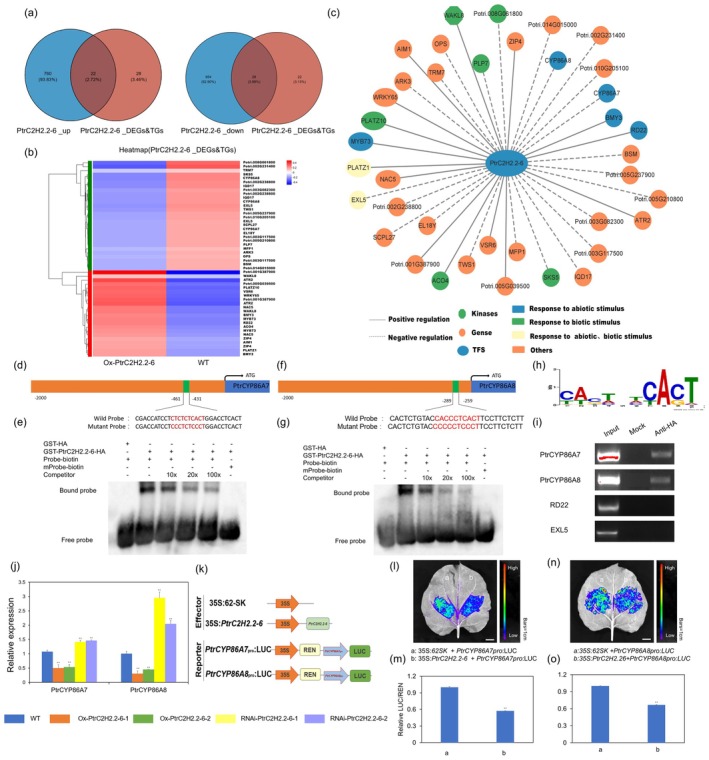
PtrC2H2.2‐6 represses the expression of cutin, suberin and wax biosynthesis key genes *PtrCYP86A7* and *PtrCYP86A8*. (a) Venn diagram of upregulated differential target genes and down‐regulated differential target genes. Blue circles denote genes that are either up‐ or down‐regulated in the Ox‐*PtrC2H2.2‐6* line relative to the wild type. Red circles indicate the predicted target genes of *PtrC2H2.2‐6* among the differentially expressed genes. (b) Heat map of differential target genes in Ox‐*PtrC2H2.2‐6* and WT. The red and blue bars indicate high and low expression, respectively. (c) Regulatory network diagram of downstream target genes regulated by *PtrC2H2.2‐6*. Solid lines represent positive regulation, and dashed lines represent negative regulation. Hexagons represent kinases, circles represent genes and ovals represent transcription factors. Blue represents response to abiotic stimulus, green represents response to biotic stimulus, yellow represents response to both abiotic and biotic stimulus and orange represents other. (d) Diagram of the wild type and mutated probes used for electrophoretic mobility shift assay (EMSA). The wild probe is a putative *PtrC2H2.2‐6* transcription factor binding site (CTCTCTCACT) on the *PtrCYP86A7* promoter. In the mutant probe, the putative binding site sequence CTCTCTCACT was replaced with CCCTCTCCCT. (e) The result of EMSA assays. The purified GST‐*PtrC2H2.2‐6*‐HA protein binds CTCTCTCACT sequences in the promoter region of *PtrCYP86A7*, and this binding is attenuated with increasing concentrations of competitive probes. GST‐HA was used as a control. (f) Diagram of the wild type and mutated probes used for electrophoretic mobility shift assay (EMSA). The wild probe is a putative *PtrC2H2.2‐6* transcription factor binding site (CACCCTCACT) on the *PtrCYP86A8* promoter. In the mutant probe, the putative binding site sequence CACCCTCACT was replaced with CCCCCTCCCT. (g) The result of EMSA assays. The purified GST‐*PtrC2H2.2‐6*‐HA protein binds CACCCTCACT sequences in the promoter region of *PtrCYP86A8*, and this binding is attenuated with increasing concentrations of competitive probes. GST‐HA was used as a control. (h) Bioinformatic analysis of PtrC2H2.2‐6 TFBS motifs. The size of the letter represents the probability of that base occurring at this position. (i) DAP‐PCR verification of predicted direct targets of PtrC2H2.2‐6. Input is fragmented plant DNA fragments; mock is the product of GST‐HA null protein eluted after incubation with fragmented plant DNA fragments; anti‐HA is the product of GST‐PtrC2H2.2‐6‐HA protein eluted after incubation with fragmented plant DNA fragments. RD22 and EXL5 are negative controls. (j) The transcript levels of *PtrCYP86A7* and *PtrCYP86A8* in leaves of 4‐week‐old WT, Ox‐*PtrC2H2.2‐6* and RNAi‐*PtrC2H2.2‐6* plants. Asterisks denote significant differences. **, *p* ≤ 0.01. (k) The schematic diagrams of the effector and reporter constructs used for a dual‐luciferase assay. Effector includes 35S:62SK and 35S:*PtrC2H2.2‐6*. The reporter includes *PtrCYP86A7pro*:LUC and *PtrCYP86A8pro*:LUC. (l) The dual‐luciferase reporter assay shows that PtrC2H2.2‐6 represses the expression of *PtrCYP86A7*. a represents co‐transformation of 35S:62SK and *PtrCYP86A7pro*:LUC in tobacco. b represents the co‐transformation of 35S:*PtrC2H2.2‐6* and *PtrCYP86A7pro*:LUC in tobacco. (m) LUC as a reporter gene and REN as an internal reference gene, LUC/REN activity detection to verify PtrC2H2.2‐6 directly represses the expression of *PtrCYP86A7*. (n) The dual‐luciferase reporter assay shows that PtrC2H2.2‐6 represses the expression of *PtrCYP86A8*. a represents the co‐transformation of 35S:62SK and *PtrCYP86A8pro*:LUC in tobacco. b represents the co‐transformation of 35S:*PtrC2H2.2‐6* and *PtrCYP86A8pro*:LUC in tobacco. (o) LUC as a reporter gene and REN as an internal reference gene, LUC/REN activity detection to verify PtrC2H2.2‐6 directly represses the expression of *PtrCYP86A8*. Values are means ± SD (*n* = 9). Asterisks denote significant differences. *, *p* ≤ 0.05; **, *p* ≤ 0.01.

Based on the transcriptional regulatory activity of *PtrC2H2.2‐6*, we integrated the downstream target genes predicted by the Plant Transcription Factor Database (TFDB) with the DEGs identified through RNA‐seq (Tables S1 and S2). The Venn diagram revealed 50 downstream target genes that may be regulated by *PtrC2H2.2‐6*, among whom 22 genes were down‐regulated and 28 genes were upregulated in overexpressing plants (Figure 5a,b; Table S3). Notably, *PtrCYP86A7* and *PtrCYP86A8*, which are implicated in the signalling pathway for cutin, suberin and wax biosynthesis, were also included. The subsequent functional regulatory network indicated that both *PtrCYP86A7* and *PtrCYP86A8* responded to abiotic stimulus and are transcriptionally repressed by *PtrC2H2.2‐6* (Figure 5c; Table S3). In addition, through MEME analysis, the DNA motif bound by PtrC2H2.2‐6 was identified as the conserved bases of CACTXTCACT (Figure 5h). In order to verify that *PtrC2H2.2‐6* directly binds to the promoters of *PtrCYP86A7* and *PtrCYP86A8*, electrophoretic mobility shift assays (EMSA) were conducted using 30 bp promoter fragments of these genes, labelled with biotin (Figure 5d,f). The purified GST‐PtrC2H2.2‐6‐HA protein bound to these labelled probes, forming visible bands that disappeared when mutant probes were used (Figure 5e,g). Competitive binding assays showed that increasing concentrations of unlabeled probes gradually weakened the binding. DAP‐PCR experiments further validated the interaction between *PtrC2H2.2‐6* and these promoter sequences (Figure 5i). Expression analyses showed that *PtrCYP86A7* and *PtrCYP86A8* transcript levels were increased in RNAi‐*PtrC2H2.2‐6* poplars and decreased in Ox‐*PtrC2H2.2‐6* poplars in contrast to the wild type (Figure 5j; Methods S2), consistent with transcriptomic data. Meanwhile, analysis of the tissue expression levels of *PtrCYP86A7/A8* revealed that they were mainly expressed in young and mature leaves, with lower expression in roots and stems (Figure S7), which happened to be opposite to the expression pattern of *PtrC2H2.2‐6* that was mainly expressed in roots and stems, with lower expression levels in young and mature leaves, which implied that *PtrC2H2.2‐6* might negatively regulate the *PtrCYP86A7/A8* expression. Furthermore, dual‐luciferase assays in *N. benthamiana* confirmed that *PtrC2H2.2‐6* represses the activities of *PtrCYP86A7* and *PtrCYP86A8* promoters (Figure 5k–o; Methods S15). Together, these findings show that *PtrC2H2.2‐6* directly binds to the promoters of *PtrCYP86A7* and *PtrCYP86A8* and suppresses their expression.

### 3.6 *PtrCYP86A7/A8* May Improve Drought Tolerance in Poplar by Regulating Leaf Surface Wax Biosynthesis

**FIGURE 6:**
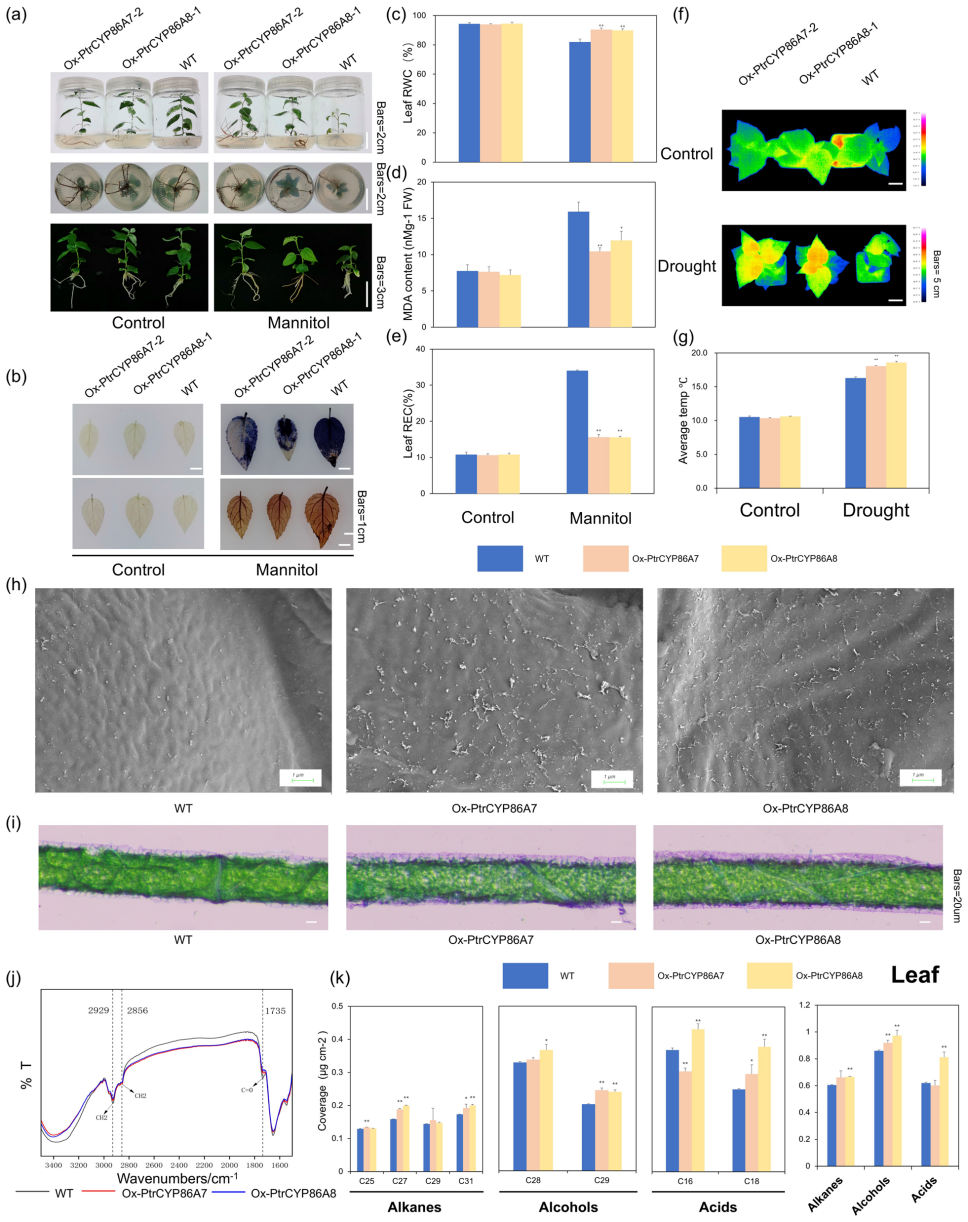
*PtrCYP86A7/A8* enhance drought tolerance by enhancing leaf surface cutin and wax synthesis. (a) The wild type (WT), Ox‐*PtrCYP86A7* and Ox‐*PtrCYP86A8* poplar plantlets were transplanted into two variations of solid Woody Plant Medium (WPM): one devoid of any supplementary substances and the other enriched with 150 mM mannitol. The growth of all poplar plantlets was consistent prior to any treatment. (b) DAB and NBT staining were used to detect O_2_^−^ and H_2_O_2_ in Ox‐*PtrCYP86A7*, Ox‐*PtrCYP86A8* and wild‐type plants in mannitol‐added and non‐mannitol‐added WPM media. Quantitative measurement of RWC (relative water content) (c), quantitative measurement of MDA content (d), and REC (relative electrical conductance) (e) in the leaves of Ox‐*PtrCYP86A7*, Ox‐*PtrCYP86A8* and wild‐type plants in mannitol‐added and non‐mannitol‐added WPM media. Values are means ± SD (*n* = 9). Asterisks denote significant differences. *, *p* ≤ 0.05; **, *p* ≤ 0.01. (f) Infrared thermography of leaf temperature in Ox‐*PtrCYP86A7*, Ox‐*PtrCYP86A8* and wild‐type poplar after drought treatment. Colour bars represent different temperatures. (g) Statistical analysis of leaf temperatures in (f). Values are means ± SD (*n* = 9). Asterisks denote significant differences. *, *p* ≤ 0.05; **, *p* ≤ 0.01. (h) SEM images of cuticular wax crystals on mature poplar leaves at the same position of 6‐week‐old Ox‐*PtrCYP86A7*, Ox‐*PtrCYP86A8* and wild‐type plants. Scale bars = 1 μm. (i) Microscope images of 6‐week‐old poplar leaves of different lines in the longitudinal section. Bars = 20 μm. (j) Fourier transform infrared spectroscopy (FTIR) of substances extracted from poplar same‐part mature leaves in different lines. The black line represents WT, the red line represents Ox‐*PtrCYP86A7* and the blue line represents Ox‐*PtrCYP86A8*. (k) Cuticular cutin and wax composition in poplar same‐part mature leaves of 4‐week‐old Ox‐*PtrCYP86A7*, Ox‐*PtrCYP86A8* and wild‐type plants. Values are means ± SD (*n* = 3). Asterisks denote significant differences. **, *p* ≤ 0.01.

In order to better comprehend *PtrCYP86A7* and *PtrCYP86A8's* functions during drought stress, we generated transgenic poplar lines overexpressing these genes, which were verified at the DNA, RNA, and protein levels (Figures S2 and S3). Subsequently, we chose one of the higher‐expressing lines, Ox‐*PtrCYP86A7‐2* and Ox‐*PtrCYP86A8‐1*, for follow‐up experiments, respectively. Drought was simulated using 100 mM mannitol in WPM medium (Methods S5). After 1 month of treatment, wild‐type plants displayed more pronounced leaf yellowing and curling compared to the overexpressing lines (Figure 6a). Additionally, plants that overexpressed the *PtrCYP86A7* and *PtrCYP86A8* genes demonstrated higher drought tolerance than wild‐type plants during short drought conditions (Figure S4; Methods S5). Besides the distinct phenotypic variations, all plant lines showed a drop in relative leaf water content after mannitol treatment. However, in contrast to the transgenic lines that overexpressed *PtrCYP86A7* and *PtrCYP86A8*, the decrease was noticeably more noticeable in wild type (Figure 6c; Methods S6). Furthermore, mannitol‐induced stress resulted in increased MDA levels and leaf relative conductance in all lines, with wild‐type plants exhibiting higher MDA content and a more substantial increase in leaf relative conductance (Figure 6d,e; Methods S6). This suggests that wild‐type plants experienced more severe oxidative stress. Histological analysis using NBT and DAB staining revealed greater accumulation of O_2_^−^ and H_2_O_2_ in wild‐type plants (Figure 6b; Methods S7). Additionally, thermal imaging indicated that leaves of overexpression plants had higher temperatures, while wild‐type plants had lower temperatures following drought treatment (Figure 6f,g; Methods S8). These findings further corroborate that wild‐type plants sustained more severe damage under drought stress conditions.

Similarly, SEM showed increased wax on the leaf surfaces of Ox‐*PtrCYP86A7* and Ox‐*PtrCYP86A8* poplars compared with wild type (Figure 6h). Staining of longitudinal sections of leaves similarly showed that Ox‐*PtrCYP86A7/A8* plants were more enriched in cuticle‐related substances compared with the wild type (Figure 6i). Furthermore, FTIR analysis demonstrated that the absorption intensities of Ox‐*PtrCYP86A7* and Ox‐*PtrCYP86A8* were larger than those of the wild type at 2929 cm^−1^ (symmetric CH_2_ telescoping vibration) and 1735 cm^−1^ (C=O telescoping vibration), which was in agreement with the previous results of RNAi‐*PtrC2H2.2‐6* that had more characteristic functional groups representing cutin and wax synthesis‐related substances (Figure 6j). GC–MS further quantified surface wax components, showing significantly elevated levels of alkanes (C25, C27 and C31) and primary alcohols (C28 and C29) in Ox‐*PtrCYP86A7/A8* plants compared with the wild type (Figure 6k). Besides, Ox‐*PtrCYP86A7* plants had a significantly higher content of C18 fatty acids and a lower content of C16 fatty acids when compared to the wild type. The levels of C16 and C18 fatty acids both increased in Ox‐*PtrCYP86A8* plants. Collectively, these findings imply that the overexpression of *PtrCYP86A7* and *PtrCYP86A8* may enhance drought tolerance in plants by increasing wax biosynthesis.

### 3.7 The Kinase PtrPPK1 Interacts With *PtrC2H2.2‐6* and Phosphorylates the PtrC2H2.2‐6 Protein, Thereby Regulating the Stability of the *PtrC2H2.2‐6*

**FIGURE 7:**
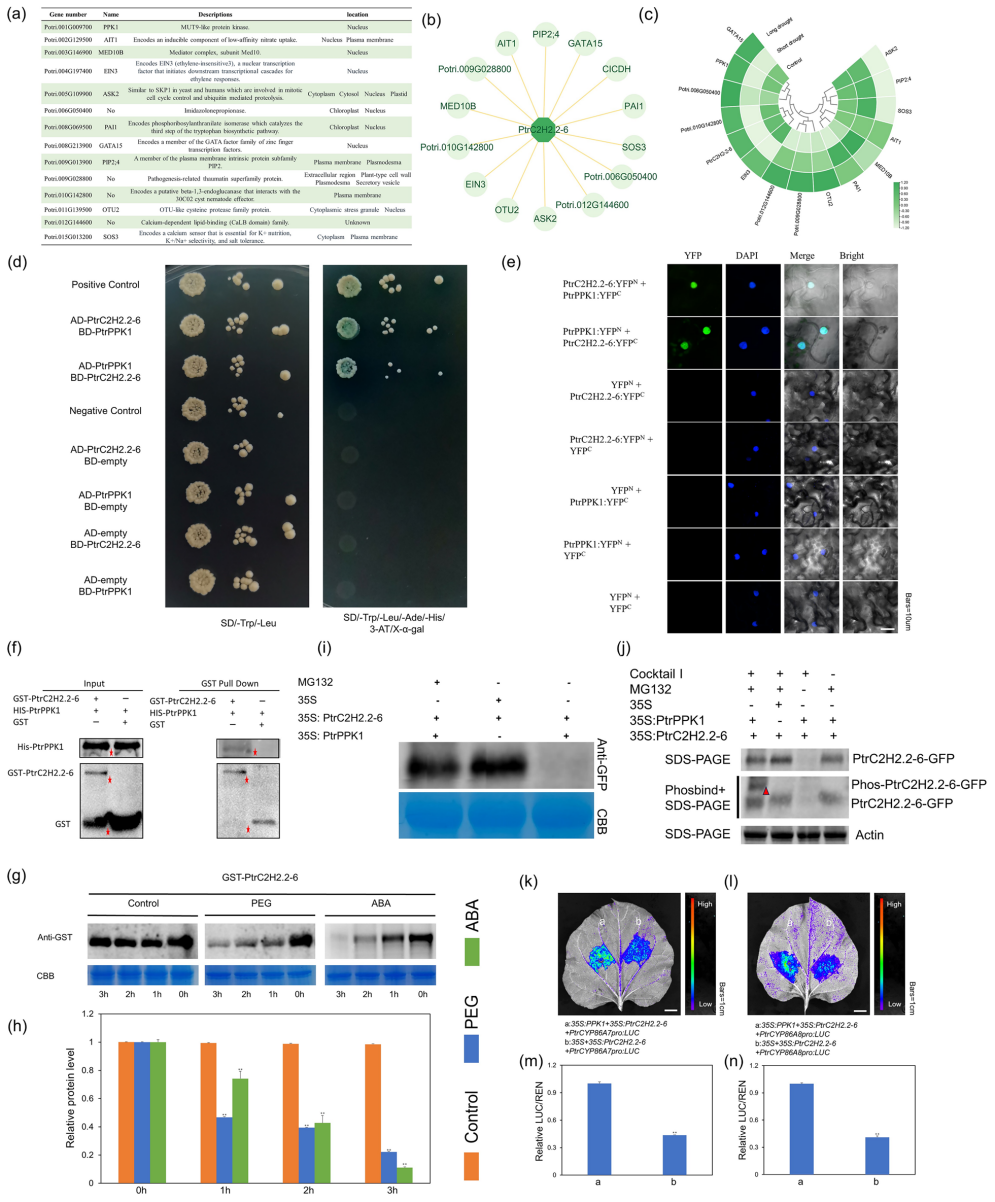
PtrPPK1‐mediated PtrC2H2.2‐6 degradation regulates *PtrCYP86A7/8* expression. (a) Functional annotation of proteins tested for interaction with PtrC2H2.2‐6 using the yeast two‐hybrid screening method. (b) Protein interaction network diagram for PtrC2H2.2‐6. Dark green represents PtrC2H2.2‐6, and light green represents interacting proteins. (c) The expression levels of *PtrC2H2.2‐6* and its interacting proteins under control, short drought and long drought. The green and white bars (FPKM) indicate high and low expression, respectively. (d) A point‐by‐point yeast two‐hybrid assay that demonstrated the interactions between PtrPPK1 and PtrC2H2.2‐6. Yeast cells were grown on SD/−T/−L and SD/−L/−T/‐H/−A media for 5 d, respectively. pGBKT7‐53 and pGADT7‐T were used as positive controls, and pGBKT7‐Lam and pGADT7‐T were used as negative controls. (e) A bimolecular fluorescence complementation (BiFC) assay was used to demonstrate the in vivo interaction between PtrC2H2.2‐6 and PtrPPK1 in the nucleus. PtrC2H2.2‐6‐YNE and PtrC2H2.2‐6‐YCE are co‐expressed with PtrPPK1‐YCE and PtrPPK1‐YNE, respectively, in *N. benthamiana* leaves. Null‐YCE and null‐YNE were used as negative controls. Bars = 10 μm. (f) In vitro GST‐pulldown assays showing the interaction between PtrC2H2.2‐6 and PtrPPK1. The protein PtrPPK1‐His was incubated with either GST or GST‐PtrC2H2.2‐6. Following incubation, a washing step was performed to eliminate any unbound proteins. Subsequently, the proteins that remained bound were eluted and subjected to immunoblotting analysis using antibodies specific to both GST and His tags. The predicted size of the His‐PtrPPK1 protein is approximately 82 kDa; the predicted size of the GST‐PtrC2H2.2‐6‐HA protein is approximately 54 kDa. (g) Degradation of the GST‐PtrC2H2.2‐6‐HA fusion protein with control (no treatment), PEG (30% PEG6000) and ABA treatments. (h) Relative protein level as indicated in (g). Values are means ± SD (*n* = 3). Asterisks denote significant differences. **, *p* ≤ 0.01. (i) PtrPPK1‐mediated degradation of PtrC2H2.2‐6 in tobacco leaves, which was inhibited by MG132. Co‐transformation of the 35S empty vector and 35S:*PtrPPK1* with 35S:*PtrC2H2.2‐6* in tobacco, respectively, the results showed that PtrPPK1 was able to induce the degradation of PtrC2H2.2‐6, and this degradation process could be inhibited by the proteasome inhibitor MG132. The predicted size of the PtrC2H2.2‐6‐GFP protein is approximately 55 kDa. (j) PtrPPK1 phosphorylates PtrC2H2.2‐6 in vivo. Co‐transformation of the 35S empty vector and 35S:PtrPPK1 with 35S:PtrC2H2.2‐6 in tobacco, respectively, the results showed that PtrPPK1 is able to phosphorylate PtrC2H2.2‐6. Cocktail I is a phosphatase inhibitor, primarily inhibiting dephosphorylation. MG132 is a proteasome inhibitor that primarily inhibits the degradation of proteins. (k) Dual luciferase reporter assays showed that PtrPPK1 relieved the inhibition of PtrC2H2.2‐6 on *PtrCYP86A7*. (l) Dual luciferase reporter assays showed that PtrPPK1 relieved the inhibition of PtrC2H2.2‐6 on PtrCYP86A8. (m) LUC/REN activity detection to verify PtrPPK1 relieved the inhibition of PtrC2H2.2‐6 on PtrCYP86A7. (n) LUC/REN activity detection to verify PtrPPK1 relieved the inhibition of PtrC2H2.2‐6 on *PtrCYP86A8*. Values are means ± SD (*n* = 9). Asterisks denote significant differences. **, *p* ≤ 0.01.

Given the lack of self‐activation in *PtrC2H2.2‐6* (Figure 1e; Methods S4), a yeast two‐hybrid screening experiment was conducted using it for bait (Figure S5; Methods S4). This analysis identified 14 potential interacting proteins (Figure 7a), allowing the construction of a protein interaction network (Figure 7b). Most of these proteins have locations in the nucleus, plasma membrane, cytoplasm and chloroplast positions, and four of them—Polyphosphate kinase 1 (*PPK1*), also known as *AEL2* and *MLK4*, ethylene‐insensitive 3 (*EIN3*), and mediator of RNA polymerase II transcription subunit 10b (*MED10B*) and GATA transcription factor 15 (*GATA15*) proteins localised to the nucleus with similar localization signals as *PtrC2H2.2‐6* (Figure 7a). Among them, the expression patterns of *GATA15*, *EIN3* and *PPK1* under drought conditions were similar to those of *PtrC2H2.2‐6* (Figure 7c; Methods S14; Table S6). Besides, RT‐qPCR results showed that the expression of *PtrPPK1* decreased significantly after drought treatment (Figure S9a), consistent with *PtrC2H2.2‐6*, suggesting that they may regulate the response of poplar to drought through protein interactions. Meanwhile, the expression of *PtrPPK1* decreased after ABA treatment (Figure S9b). In addition, it was shown that *PPK1* and its homologous genes (*AEL1‐4*) are insensitive to ABA but can be induced to be expressed by ABA (H. Chen et al. 2018). This is in contrast to the expression pattern of *PtrPPK1* in poplar, and it is possible that they are functionally differentiated. Consistent with the above hypothesis, we overexpressed the *PtrPPK1* gene in poplar and confirmed that PtrPPK1 positively regulates drought resistance in poplar (Figure S14). Additionally, we found that, compared with wild‐type plants, genes related to ROS (*RBOHD/F*) and ABA (*ABI5/3* and *ABF3*) signal transduction were significantly upregulated in poplar overexpressing *PtrPPK1*. Therefore, *PtrPPK1* may be involved in the plant's response to drought by participating in ABA and ROS signal transduction (Figure S15). Also, given previous reports linking *PPK1* to ABA signalling and osmotic stress (Wang et al. 2015; Chen et al. 2018), we postulated that PtrPPK1 interacts with PtrC2H2.2‐6 to mediate drought tolerance in poplar.

Initially, we conducted a yeast peer‐to‐peer validation experiment (Methods S4). The findings demonstrated that transformants containing the AD‐*PtrC2H2.2‐6* and BD‐*PtrPPK1* constructs, as well as those with the AD‐*PtrPPK1* and BD‐*PtrC2H2.2‐6* constructs, were able to grow on screening medium that lacked Trp, Leu, Ade and His (Figure 7d). In contrast, the control did not exhibit growth on this screening medium. Besides, Bifc experiments conducted in tobacco leaves revealed that YNE‐*PtrPPK1* and YCE‐*PtrC2H2.2‐6*, as well as YCE‐*PtrPPK1* and YNE‐*PtrC2H2.2‐6*, were able to detect fluorescent signals in the nucleus, while none of the control samples exhibited such signals (Figure 7e; Methods S16). This is consistent with the message that *PtrPPK1* is localised in the nucleus (Figure S6; Methods S3). Furthermore, the interactions were corroborated through in vitro GST‐pulldown experiments, demonstrating that PtrC2H2.2‐6‐GST could directly bind to PtrPPK1‐His (Figure 7f). Collectively, these results confirm a direct interaction between PtrC2H2.2‐6 and PtrPPK1 within the nucleus.

In addition, we explored whether *PtrPPK1* is transcriptionally regulated by *PtrC2H2.2‐6*. To this end, we carefully analysed the promoter region of *PtrPPK1* and, unfortunately, did not find the most conserved binding motif of *PtrC2H2.2‐6* in its promoter region (Figure 5h). Meanwhile, we also examined the expression of *PtrPPK1* in different transgenic plants (including Ox‐*PtrC2H2.2‐6* and RNAi‐*PtrC2H2.2‐6* plants), and both RT‐qPCR and transcriptome results indicated that its expression level did not differ significantly among different plants (Figure S10). These results suggest that, at the transcriptional level, *PtrPPK1* is not regulated by *PtrC2H2.2‐6*.

To assess whether drought influences the stability of PtrC2H2.2‐6, we employed PEG to simulate drought in plants and tested the protein's stability at several time periods. Results showed that PEG administration increased the degradation of PtrC2H2.2‐6 protein compared to the control (Figure 7g,h), indicating that drought regulates *PtrC2H2.2‐6* at both the transcriptional and protein levels. Meanwhile, we found that compared with the control group, treatment with ABA also accelerated the degradation of PtrC2H2.2‐6 protein (Figure 7g,h), which means that ABA also disrupts the stability of PtrC2H2.2‐6 at the protein level. Given that *PtrPPK1* is a phosphokinase, it phosphorylates *HY5* and promotes its binding to the ubiquitin ligase *COP1*, which in turn leads to its degradation (Zhang et al. 2024); we hypothesised it may mediate *PtrC2H2.2‐6* degradation. Co‐expression of 35S:*PtrPPK1* and 35S:*PtrC2H2.2‐6* in tobacco leaves confirmed that PtrPPK1 accelerates PtrC2H2.2‐6 degradation, a process inhibited by the proteasome inhibitor MG132 (Figure 7i). This suggests that *PtrPPK1* might influence *PtrC2H2.2‐6* degradation via the 26S ubiquitin pathway. Moreover, phosphorylation assays provided additional insights into the functional interaction within the PtrPPK1‐PtrC2H2.2‐6 module. Notably, the presence of 35S:PtrPPK1 promoted the formation of the phosphorylated PtrC2H2.2‐6‐GFP band (Figure 7j; Methods S17), thus confirming the ability of PtrPPK1 to phosphorylate PtrC2H2.2‐6. Since this is an in vivo phosphorylation assay, we simulated the dephosphorylation effect by adding phosphatase or not adding the phosphatase inhibitor (Cocktail I). The experimental results showed that the phosphorylation band of PtrC2H2.2‐6‐GFP disappeared when phosphatase was added or Cocktail I was not added (Figure 7j and Figure S13). Furthermore, Dual‐luciferase reporter experiments were conducted to examine whether *PtrPPK1* mediates the regulation of *PtrCYP86A7* and *PtrCYP86A8* expression by *PtrC2H2.2‐6*. The findings demonstrated the co‐expression of 35S:*PtrPPK1* and 35S:*PtrC2H2.2‐6* significantly alleviated the repression of *PtrCYP86A7* and *PtrCYP86A8* by *PtrC2H2.2‐6* alone, resulting in elevated reporter activity (Figure 7k–n; Methods S14). This indicates that *PtrPPK1* mitigates the inhibitory effect of *PtrC2H2.2‐6* on these downstream genes by promoting its degradation.

## 4 Discussion

Cutin and wax, essential parts of the plant cuticle, are crucial for mitigating abiotic stresses by reducing transpiration, which is critical for drought tolerance (Yang et al. 2011; Xue et al. 2017). Memberships of the CYP86A family, including *CYP86A1*, *CYP86A2*, *CYP86A4*, *CYP86A7* and *CYP86A8*, have distinct roles in the cutin, suberin and wax biosynthesis pathway (Duan and Schuler 2005). For instance, *AtCYP86A1* is the first fatty acid X‐monooxygenase identified in plants, which is involved in the hydroxylation of C16–C18 long‐chain fatty acids (Höfer et al. 2008). In addition, *CYP86A4/7* in Arabidopsis and *OsCYP86A9* in rice can respectively promote the deposition of cutin and suberin in plants (Kannangara et al. 2007; Huang et al. 2024).

In this study, some evidence supports that *PtrCYP86A7/A8* can affect the structure of the leaf cuticle in poplar. Furthermore, scanning electron microscopy of leaves and toluidine blue staining respectively showed that *PtrCYP86A7/A8* can increase the accumulation of cutin and wax. In addition, studies have shown that *GbCYP86A1‐1* can increase the content of C16–C18 fatty acids in plants (Wang et al. 2019). Consistent with the aforementioned reports, the relative content of C16–C18 long‐chain fatty acids in the leaves of Ox‐*PtrCYP86A7/A8* poplar trees was significantly increased, indicating that there were sufficient precursor substances for the synthesis of cutin and wax. Additionally, the wax metabolome analysis showed that the content of C25, C27 and C31 alkanes, as well as C28 and C29 primary alcohols, was higher in Ox‐*PtrCYP86A7/A8* plants compared to the WT. These compounds are closely related to the formation of wax in poplar (He et al. 2022).

Under drought conditions, plants mitigate non‐stomatal water loss by enhancing leaf cuticle accumulation (Kosma et al. 2009; Seo et al. 2011). Consequently, in contrast to the wild type, the Ox‐PtrCYP86A7/A8 poplar under drought stress exhibited a greater relative leaf water content. After all, cuticular transpiration is crucial for regulating plant water balance and temperature constancy (Bueno et al. 2019). This idea is supported by a number of studies; for example, the abundance of long‐chain alkane and ester components in the epidermal waxes of rye leaves affects their hydrophobicity, which in turn leads to significantly higher relative water content of the leaves of waxed varieties than those of unwaxed varieties under drought stress (Laskos et al. 2021). At the same time, the increase of esters in banana leaf wax was positively correlated with the maintenance of relative water content of banana leaves (Sampangi‐Ramaiah et al. 2016). This suggests that the wax composition of the leaf surface is closely related to the relative water content of the leaf. In line with these findings, the Ox‐*PtrCYP86A7/A8* leaves' surface temperature was noticeably higher compared with the wild type's under drought conditions, likely due to improved water retention from increased cutin and wax content.

Although our understanding of plant cuticle biosynthesis is advancing, the intricate regulatory mechanisms remain largely elusive. Specifically, research on the upstream transcriptional regulation of the CYP86A family is limited, with only the transcription factor *WIN1*, for instance, regulating *CYP86A4* and *CYP86A7* expression, enhancing cuticle formation in Arabidopsis (Kannangara et al. 2007). To date, there have been almost no studies on the upstream regulatory transcription factors of *PtrCYP86A7/8*. In this study, a variety of biochemical experiments and bioinformatics analyses indicated that *PtrCYP86A7/A8* is a direct downstream target gene of *PtrC2H2.2‐6*, and the promoter region of *PtrCYP86A7/A8* contains a binding site (CACT) for the transcription factor *PtrC2H2.2‐6*.

Furthermore, *PtrC2H2.2‐6* negatively regulates the expression of *PtrCYP86A7/A8*, thereby reducing the synthesis of cutin and wax. However, some studies have shown that certain C2H2 transcription factors in plants (e.g., *CsGLF1* and *CsDULL* in cucumber) positively regulate the accumulation of plant waxes (Zhai et al. 2022; Yang et al. 2023). This is largely likely to be caused by the functional divergence of C2H2 transcription factors during the evolutionary process (Zhao, Liu, et al. 2024). Now, we observed that poplar plants with silenced *PtrC2H2.2‐6* and those overexpressing *PtrCYP86A7* and *PtrCYP86A8* exhibited similar drought‐resistant phenotypes and cuticle structures. Consistent with the aforementioned results, *PtrC2H2.2‐6* is expressed at low levels in leaves, while its target gene *PtrCYP86A7/A8* is highly expressed in leaves (Figure 1a and Figure S8), which may also facilitate the normal participation of poplar leaves in the synthesis of cutin and wax. However, when compared individually with the WT poplars, the trends in the changes of C16 and C18 fatty acid content in RNAi‐*PtrC2H2.2‐6* and Ox‐*PtrCYP86A7/A8* plants are not consistent. Firstly, C16 and C18 fatty acids serve as the initial substrates for the synthesis of cutin and wax, while *CYP86A7/A8* acts as the first enzyme in the biosynthetic pathway of cutin and wax (Zhao et al. 2025). This is also the main reason for the differences in C16 and C18 fatty acids in the leaves of RNAi‐*PtrC2H2.2‐6* and Ox‐*PtrCYP86A7/A8* plants. Secondly, the wax biosynthesis pathway involves the coordinated action of multiple genes. *PtrC2H2.2‐6* may indirectly suppress other genes related to wax synthesis (including *CER* and *KCS*), thereby indirectly affecting C16 and C18 fatty acid content. This study qualitatively investigated that the *PtrC2H2.2‐6*—*PtrCYP86A7/A8* module does indeed affect the formation of cutin and wax in poplar leaves. Due to the difficulty in extracting cutin from poplar leaves, this study only quantitatively analysed the content of metabolites related to wax monomers at the quantitative level, and the quantitative analysis of cutin monomers in the poplar leaves of this module will be the direction of our future research.

Transcription factors of the C2H2 type are commonly found in plants and are essential for their response to drought stress, influencing plant drought resistance by regulating a range of biological processes (Liu et al. 2022; Razin et al. 2012). For instance, *MdZAT10* in apples negatively regulates drought tolerance by promoting ROS accumulation (Yang, Li, et al. 2021), while overexpression of *ZPT2‐3* in petunia enhances drought tolerance (Sugano et al. 2003). Our analysis revealed reduced *PtrC2H2.2‐6* expression at both the transcript and protein levels under drought conditions, aligning with its role in negatively regulating drought responses. Consistent with the aforementioned results, ABA treatment also accelerated the destabilisation of the PtrC2H2.2‐6 protein. However, under ABA treatment, PtrC2H2.2‐6 showed inconsistency between the protein level and the transcriptional level, and we speculated that this was likely due to its own feedback regulatory mechanisms or post‐translational modifications of the protein (Waadt et al. 2022). In this study, a large number of protein interaction experiments have confirmed that PtrC2H2.2‐6 can interact with the protein kinase PtrPPK1. In Arabidopsis, PPK1 is a protein kinase that participates in stress resistance by regulating protein stability (Wang et al. 2015; Chen et al. 2018). For instance, in Arabidopsis, *PPK1* phosphorylates *HY5*, promoting its degradation by *COP1* (Zhang et al. 2024). Similarly, biochemical experiments have confirmed that PtrPPK1 can phosphorylate and modify the PtrC2H2.2‐6 protein, leading to the degradation of the PtrC2H2.2‐6 protein through the 26S ubiquitin‐proteasome pathway. Furthermore, based on our experimental results, we boldly speculate that the degradation of PtrC2H2.2‐6 by PtrPPK1 alleviates the inhibition of *PtrCYP86A7/A8* expression by *PtrC2H2.2‐6*, thereby enhancing the plant's drought resistance.

**FIGURE 8:**
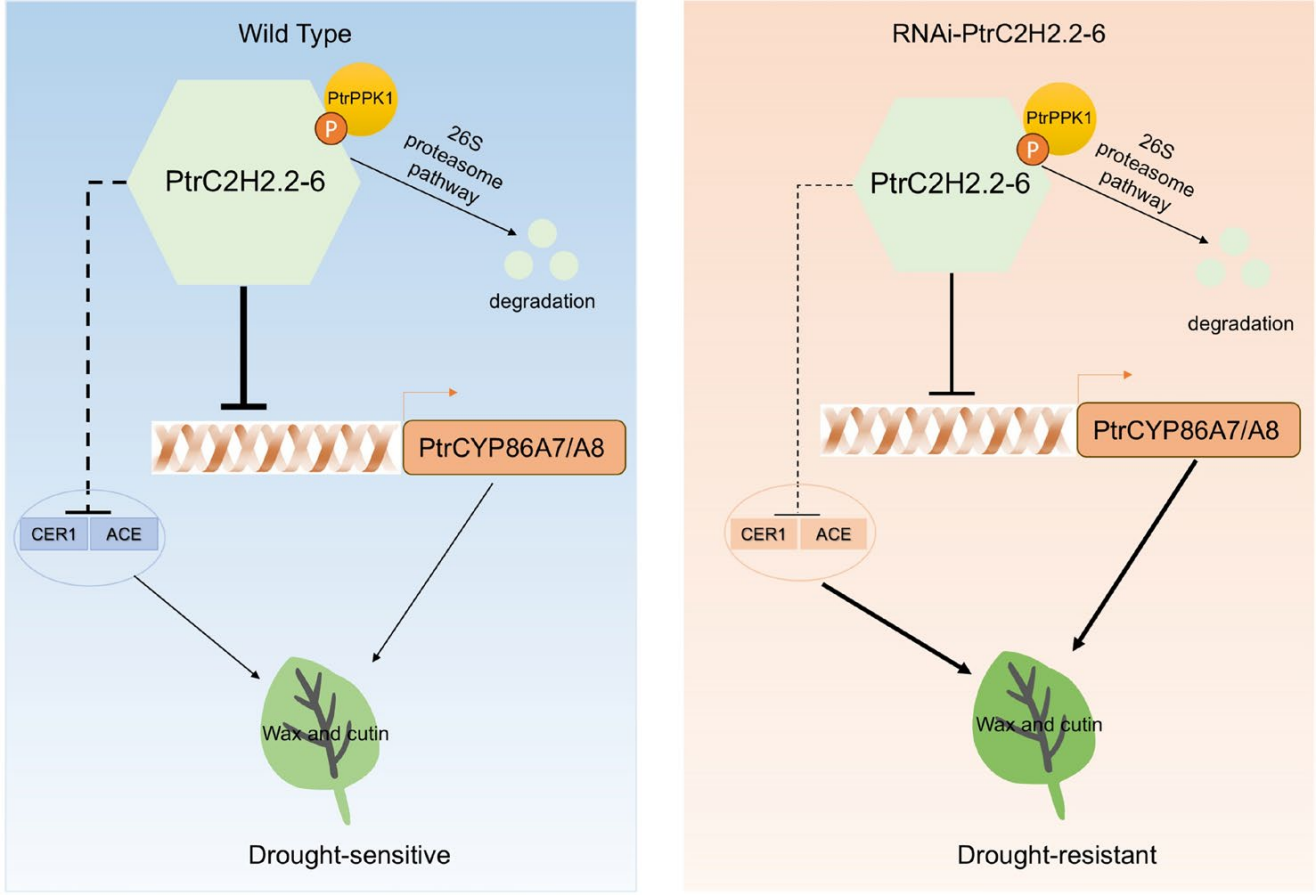
Modelling of RNAi‐*PtrC2H2.2‐6* and Wild type regulation of drought tolerance in plants. The critical role of the *PtrPPK1*‐*PtrC2H2.2‐6*‐*PtrCYP86A7/A8* module in regulating drought tolerance in poplar. Under drought stress, the RNAi‐*PtrC2H2.2‐6* poplar lines displayed improved drought resistance, contrasting with the increased susceptibility observed in the Ox‐*PtrC2H2.2‐6* lines. *PtrC2H2.2‐6* was found to negatively regulate the biosynthesis of cutin and wax, thereby affecting leaf surface wax formation and the plant's overall drought resistance. At the molecular level, *PtrC2H2.2‐6* suppresses the expression of *PtrCYP86A7/8* by binding to CACT motifs in their promoters. Additionally, PtrPPK1 interacts with PtrC2H2.2‐6, phosphorylates it, and promotes its degradation, thereby relieving its repression of PtrCYP86A7/8. Furthermore, we discovered that the overexpression of *PtrCYP86A7* and *PtrCYP86A8* in poplar enhances the production of metabolic products associated with cutin and wax biosynthesis, which in turn promotes leaf surface wax formation and improves drought resistance. Collectively, the *PtrPPK1‐PtrC2H2.2‐6‐PtrCYP86A7/A8* module is central to the regulation of poplar's response to drought stress through the modulation of cutin and wax biosynthesis.

This study highlights the critical role of the *PtrPPK1*‐*PtrC2H2.2‐6*‐*PtrCYP86A7/A8* module in regulating poplar's ability to withstand drought (Figure 8). By regulating the production of cutin and wax, this regulatory network governs cuticle formation and non‐stomatal water loss. Our research sheds light on the transcriptional and post‐translational control of cuticle biosynthesis, which helps develop methods to enhance woody plant tolerance to drought.

## Author Contributions

The initial research project was designed and carried out by Jiu‐Jiu Zhao, Xiang Xiang, Peng Yang, Hao Li, Ting Wang and Fang He. Jiu‐Jiu Zhao, Jin Li, Shu‐Ying Wei, Rui‐Quan Wang, Shuang‐lian Deng and Yuan Zhang conducted the experiments. Ting Wang, Jinliang Huang, Liang‐Hua Chen, HanBo Yang, Xue‐Qin Wan and Fang He designed the experiments and conducted the data analysis. Jiu‐Jiu Zhao and Fang He crafted the manuscript. Fang He modified the writing and directed the experiments. Fang He acquired the funding for the research project. The complete manuscript was read and authorised by all writers.

## Conflicts of Interest

The authors declare no conflicts of interest.

## Supporting information


**Figure S1:** GCMS‐related data.
**Figure S2:** Identifying *PtrCYP86A7*‐overexpressing transgenic poplars.
**Figure S3:** Identifying *PtrCYP86A8*‐overexpressing transgenic poplars.
**Figure S4:** Morphological differences between Ox‐*PtrCYP86A7*, Ox‐*PtrCYP86A8* and the wild type in short‐term drought assays.
**Figure S5:** Y2H screening procedure using PtrC2H2.2‐6 as a bait protein.
**Figure S6:** pbi70419‐sup‐0001‐Supinfo.docx. *PtrPPK1* is localised in the nucleus.
**Figure S7:** Tissue expression pattern of *PtrCYP86A7/A8* in *P. trichocarpa* .
**Figure S8:** Absolute expression level (TPM for RNA‐seq) of genes related to cutin and wax synthesis in the PtrC2H2.2‐6 overexpression poplar vs. WT group.
**Figure S9:** Relative expression of *PtrPPK1* following drought and ABA treatment.
**Figure S10:** The expression of *PtrPPK1* in different plants.
**Figure S11:** Phenotypic characterisation and statistics of different lines under prolonged drought conditions.
**Figure S12:** Co‐expression network diagram of genes involved in the wax, suberin and cutin synthesis pathway with *PtrC2H2.2‐6* and other transcription factors.
**Figure S13:** PtrPPK1 phosphorylates PtrC2H2.2‐6 in vivo.
**Figure S14:** Overexpression of PtrPPK1 in poplar enhances plant resistance to drought stress.
**Figure S15:** The relative expression level of genes related to ROS and ABA signal transduction in the PtrPPK1 overexpression poplar vs. WT group.
**Methods S1:** Plant growth conditions and treatments.
**Methods S2:** RNA extraction and RT‐qPCR.
**Methods S3:** Subcellular localization.
**Methods S4:** Y2H‐seq, self‐activation detection and Y2H assay.
**Methods S5:** Drought and mannitol treatments.
**Methods S6:** Measurement and analysis of physiological indicators.
**Methods S7:** DAB and NBT staining.
**Methods S8:** Thermal Imaging.
**Methods S9:** Analysis of chlorophyll fluorescence.
**Methods S10:** Leaf pre‐processing for SEM photography.
**Methods S11:** Observation of leaf longitudinal section staining.
**Methods S12:** Leaf pre‐processing for GC–MS.
**Methods S13:** Transcriptome analysis.
**Methods S14:** Expression patterns of *PtrC2H2.2‐6* and its reciprocal proteins following drought stress.
**Methods S15:** Dual luciferase reporter assay.
**Methods S16:** Bimolecular fluorescence complementation assay.
**Methods S17:** Phosphorylation experiments.

**Table S1:** DEGs in the *PtrC2H2.2‐6* overexpression poplar vs. WT group.
**Table S2:** PlantTFDB predicted downstream target genes of *PtrC2H2.2‐6*.
**Table S3:** Predicted downstream target genes in DEGs and their functional annotation.
**Table S4:** Genes involved in cutin, suberin and wax biosynthesis.
**Table S5:** All RT‐qPCR primer sequences used.
**Table S6:** Expression of *PtrC2H2.2* and its interacting proteins under drought stress in poplar.
**Table S7:** KEGG enrichment analysis statistic table.
**Table S8:** Summary of GC–MS data.

## Data Availability

The data that support the findings of this study are openly available in Dryad at https://datadryad.org/submission/377113.

## References

[pbi70419-bib-0001] Arya, G. C. , S. Sarkar , E. Manasherova , A. Aharoni , and H. Cohen . 2021. “The Plant Cuticle: An Ancient Guardian Barrier Set Against Long‐Standing Rivals.” Frontiers in Plant Science 12. 10.3389/fpls.2021.663165 PMC8267416 34249035

[pbi70419-bib-0002] Bhanot, V. , S. V. Fadanavis , and J. Panwar . 2021. “Revisiting the Architecture, Biosynthesis and Functional Aspects of the Plant Cuticle: There Is More Scope.” Environmental and Experimental Botany 183: 104364.

[pbi70419-bib-0003] Bueno, A. , A. Alfarhan , K. Arand , et al. 2019. “Effects of Temperature on the Cuticular Transpiration Barrier of Two Desert Plants With Water‐Spender and Water‐Saver Strategies.” Journal of Experimental Botany 70, no. 5: 1613–1625. 30715440 10.1093/jxb/erz018 PMC6416792

[pbi70419-bib-0004] Chen, C. , H. Chen , Y. Zhang , et al. 2020. “TBtools: An Integrative Toolkit Developed for Interactive Analyses of Big Biological Data.” Molecular Plant 13, no. 8: 1194–1202. 32585190 10.1016/j.molp.2020.06.009

[pbi70419-bib-0005] Chen, H.‐H. , L. Qu , Z.‐H. Xu , J.‐K. Zhu , and H.‐W. Xue . 2018. “EL1‐Like Casein Kinases Suppress ABA Signaling and Responses by Phosphorylating and Destabilizing the ABA Receptors PYR/PYLs in Arabidopsis.” Molecular Plant 11, no. 5: 706–719. 29505832 10.1016/j.molp.2018.02.012

[pbi70419-bib-0006] Duan, H. , and M. A. Schuler . 2005. “Differential Expression and Evolution of the Arabidopsis CYP86A Subfamily.” Plant Physiology 137, no. 3: 1067–1081. 15709153 10.1104/pp.104.055715 PMC1065407

[pbi70419-bib-0007] Greer, S. , M. Wen , D. Bird , et al. 2007. “The Cytochrome P450 Enzyme CYP96A15 Is the Midchain Alkane Hydroxylase Responsible for Formation of Secondary Alcohols and Ketones in Stem Cuticular Wax of Arabidopsis.” Plant Physiology 145, no. 3: 653–667. 17905869 10.1104/pp.107.107300 PMC2048791

[pbi70419-bib-0008] He, F. , H.‐G. Li , J.‐J. Wang , et al. 2019. “PeSTZ1, a C2H2‐Type Zinc Finger Transcription Factor From Populus Euphratica, Enhances Freezing Tolerance Through Modulation of ROS Scavenging by Directly Regulating Pe2.” Plant Biotechnology Journal 17, no. 11: 2169–2183. 30977939 10.1111/pbi.13130 PMC6790368

[pbi70419-bib-0009] He, F. , H.‐L. Wang , H.‐G. Li , et al. 2018. “PeCHYR1, a Ubiquitin E3 Ligase From Populus Euphratica, Enhances Drought Tolerance via ABA‐Induced Stomatal Closure by ROS Production in *Populus* .” Plant Biotechnology Journal 16, no. 8: 1514–1528. 29406575 10.1111/pbi.12893 PMC6041450

[pbi70419-bib-0010] He, J. , C. Li , N. Hu , et al. 2022. “ECERIFERUM1‐6A Is Required for the Synthesis of Cuticular Wax Alkanes and Promotes Drought Tolerance in Wheat.” Plant Physiology 190, no. 3: 1640–1657. 36000923 10.1093/plphys/kiac394 PMC9614490

[pbi70419-bib-0011] Heredia, A. 2003. “Biophysical and Biochemical Characteristics of Cutin, a Plant Barrier Biopolymer.” Biochimica et Biophysica Acta (BBA) ‐ General Subjects 1620, no. 1: 1–7. 12595066 10.1016/s0304-4165(02)00510-x

[pbi70419-bib-0012] Höfer, R. , I. Briesen , M. Beck , F. Pinot , L. Schreiber , and R. Franke . 2008. “The Arabidopsis Cytochrome P450 CYP86A1 Encodes a Fatty Acid ω‐Hydroxylase Involved in Suberin Monomer Biosynthesis.” Journal of Experimental Botany 59, no. 9: 2347–2360. 18544608 10.1093/jxb/ern101 PMC2423664

[pbi70419-bib-0013] Huang, X. , Y. Li , Z. Chang , et al. 2024. “Regulation by Distinct MYB Transcription Factors Defines the Roles of OsCYP86A9 in Anther Development and Root Suberin Deposition.” Plant Journal 118, no. 6: 1972–1990. 10.1111/tpj.16722 38506334

[pbi70419-bib-0014] Jian, L. , K. Kang , Y. Choi , M. C. Suh , and N.‐C. Paek . 2022. “Mutation of OsMYB60 Reduces Rice Resilience to Drought Stress by Attenuating Cuticular Wax Biosynthesis.” Plant Journal 112, no. 2: 339–351. 10.1111/tpj.15947 35984735

[pbi70419-bib-0015] Kannangara, R. , C. Branigan , Y. Liu , et al. 2007. “The Transcription Factor WIN1/SHN1 Regulates Cutin Biosynthesis in *Arabidopsis thaliana* .” Plant Cell 19, no. 4: 1278–1294. 17449808 10.1105/tpc.106.047076 PMC1913754

[pbi70419-bib-0016] Khan, S. A. , S. B. Khan , L. U. Khan , A. Farooq , K. Akhtar , and A. M. Asiri . 2018. “Fourier Transform Infrared Spectroscopy: Fundamentals and Application in Functional Groups and Nanomaterials Characterization.” In Handbook of Materials Characterization, 317–344. Springer International Publishing.

[pbi70419-bib-0017] Kosma, D. K. , B. Bourdenx , A. Bernard , et al. 2009. “The Impact of Water Deficiency on Leaf Cuticle Lipids of Arabidopsis.” Plant Physiology 151, no. 4: 1918–1929. 19819982 10.1104/pp.109.141911 PMC2785987

[pbi70419-bib-0018] Laskos, K. , I. M. Czyczyło‐Mysza , M. Dziurka , et al. 2021. “Correlation Between Leaf Epicuticular Wax Composition and Structure, Physio‐Biochemical Traits and Drought Resistance in Glaucous and Non‐Glaucous Near‐Isogenic Lines of Rye.” Plant Journal 108, no. 1: 93–119. 10.1111/tpj.15428 PMC9291005 34288188

[pbi70419-bib-0019] Lee, S. B. , and M. C. Suh . 2013. “Recent Advances in Cuticular Wax Biosynthesis and Its Regulation in Arabidopsis.” Molecular Plant 6, no. 2: 246–249. 23253604 10.1093/mp/sss159

[pbi70419-bib-0020] Lee, S. B. , and M. C. Suh . 2022. “Regulatory Mechanisms Underlying Cuticular Wax Biosynthesis.” Journal of Experimental Botany 73, no. 9: 2799–2816. 35560199 10.1093/jxb/erab509

[pbi70419-bib-0021] Li, W. , Z. Zhu , M. Chern , et al. 2017. “A Natural Allele of a Transcription Factor in Rice Confers Broad‐Spectrum Blast Resistance.” Cell 170, no. 1: 114–126.e5. 28666113 10.1016/j.cell.2017.06.008

[pbi70419-bib-0022] Li‐Beisson, Y. , M. Pollard , V. Sauveplane , F. Pinot , J. Ohlrogge , and F. Beisson . 2009. “Nanoridges That Characterize the Surface Morphology of Flowers Require the Synthesis of Cutin Polyester.” Proceedings of the National Academy of Sciences 106, no. 51: 22008–22013. 10.1073/pnas.0909090106 PMC2788479 19959665

[pbi70419-bib-0023] Lin, Y.‐C. , W. Li , Y.‐H. Sun , et al. 2013. “SND1 Transcription Factor–Directed Quantitative Functional Hierarchical Genetic Regulatory Network in Wood Formation in *Populus trichocarpa* .” Plant Cell 25, no. 11: 4324–4341. 24280390 10.1105/tpc.113.117697 PMC3875721

[pbi70419-bib-0024] Liu, Y. , A. R. Khan , and Y. Gan . 2022. “C2H2 Zinc Finger Proteins Response to Abiotic Stress in Plants.” International Journal of Molecular Sciences 23, no. 5: 2730. 35269875 10.3390/ijms23052730 PMC8911255

[pbi70419-bib-0025] Nawrath, C. 2006. “Unraveling the Complex Network of Cuticular Structure and Function.” Current Opinion in Plant Biology 9, no. 3: 281–287. 16580871 10.1016/j.pbi.2006.03.001

[pbi70419-bib-0026] Razin, S. , V. Borunova , O. Maksimenko , and O. Kantidze . 2012. “Cys2His2 Zinc Finger Protein Family: Classification, Functions, and Major Members.” Biochemistry 77: 217–226. 22803940 10.1134/S0006297912030017

[pbi70419-bib-0027] Sampangi‐Ramaiah, M. H. , K. V. Ravishankar , S. K. Seetharamaiah , et al. 2016. “Barrier Against Water Loss: Relationship Between Epicuticular Wax Composition, Gene Expression and Leaf Water Retention Capacity in Banana.” Functional Plant Biology 43, no. 6: 492–501. 32480479 10.1071/FP15296

[pbi70419-bib-0028] Seo, P. J. , S. B. Lee , M. C. Suh , M.‐J. Park , Y. S. Go , and C.‐M. Park . 2011. “The MYB96 Transcription Factor Regulates Cuticular Wax Biosynthesis Under Drought Conditions in Arabidopsis.” Plant Cell 23, no. 3: 1138–1152. 21398568 10.1105/tpc.111.083485 PMC3082259

[pbi70419-bib-0029] Shaheenuzzamn, M. , S. Shi , K. Sohail , et al. 2021. “Regulation of Cuticular Wax Biosynthesis in Plants Under Abiotic Stress.” Plant Biotechnology Reports 15: 1–12.

[pbi70419-bib-0030] Shannon, P. , A. Markiel , O. Ozier , et al. 2003. “Cytoscape: A Software Environment for Integrated Models of Biomolecular Interaction Networks.” Genome Research 13, no. 11: 2498–2504. 14597658 10.1101/gr.1239303 PMC403769

[pbi70419-bib-0031] Shepherd, T. , and D. Wynne Griffiths . 2006. “The Effects of Stress on Plant Cuticular Waxes.” New Phytologist 171, no. 3: 469–499. 16866954 10.1111/j.1469-8137.2006.01826.x

[pbi70419-bib-0032] Song, Q. , L. Kong , X. Yang , et al. 2022. “PtoMYB142, a Poplar R2R3‐MYB Transcription Factor, Contributes to Drought Tolerance by Regulating Wax Biosynthesis.” Tree Physiology 42, no. 10: 2133–2147. 35640137 10.1093/treephys/tpac060

[pbi70419-bib-0033] Sugano, S. , H. Kaminaka , Z. Rybka , et al. 2003. “Stress‐Responsive Zinc Finger Gene ZPT2‐3 Plays a Role in Drought Tolerance in Petunia.” Plant Journal 36, no. 6: 830–841. 10.1046/j.1365-313x.2003.01924.x 14675448

[pbi70419-bib-0034] Tian, F. , D.‐C. Yang , Y.‐Q. Meng , J. Jin , and G. Gao . 2020. “PlantRegMap: Charting Functional Regulatory Maps in Plants.” Nucleic Acids Research 48, no. D1: D1104–D1113. 31701126 10.1093/nar/gkz1020 PMC7145545

[pbi70419-bib-0035] Waadt, R. , C. A. Seller , P. K. Hsu , Y. Takahashi , S. Munemasa , and J. I. Schroeder . 2022. “Plant Hormone Regulation of Abiotic Stress Responses.” Nature Reviews. Molecular Cell Biology 23, no. 10: 680–694. 35513717 10.1038/s41580-022-00479-6 PMC9592120

[pbi70419-bib-0036] Wang, G. , J. Xu , L. Li , et al. 2019. “GbCYP86A1‐1 From *Gossypium barbadense* Positively Regulates Defence Against Verticillium Dahliae by Cell Wall Modification and Activation of Immune Pathways.” Plant Biotechnology Journal 18, no. 1: 222–238. 31207065 10.1111/pbi.13190 PMC6920168

[pbi70419-bib-0037] Wang, X. , Y. Guan , D. Zhang , X. Dong , L. Tian , and L. Q. Qu . 2017. “A β‐Ketoacyl‐CoA Synthase Is Involved in Rice Leaf Cuticular Wax Synthesis and Requires a CER2‐LIKE Protein as a Cofactor.” Plant Physiology 173, no. 2: 944–955. 27913740 10.1104/pp.16.01527 PMC5291035

[pbi70419-bib-0038] Wang, Z. , J. A. Casas‐Mallano , J. Xu , J.‐J. M. Riethoven , C. Zhang , and H. Cerutti . 2015. “Osmotic Stress Induces Phosphorylation of Histone H3 at Threonine 3 in Pericentromeric Regions of *Arabidopsis thaliana* .” Proceedings of the National Academy of Sciences of the United States of America 112, no. 27: 8487–8492. 26100864 10.1073/pnas.1423325112 PMC4500206

[pbi70419-bib-0039] Wu, Q. , X. Zhang , M. Peirats‐Llobet , et al. 2016. “Ubiquitin Ligases RGLG1 and RGLG5 Regulate Abscisic Acid Signaling by Controlling the Turnover of Phosphatase PP2CA.” Plant Cell 28, no. 9: 2178–2196. 27577789 10.1105/tpc.16.00364 PMC5059804

[pbi70419-bib-0040] Xiao, F. , S. Mark Goodwin , Y. Xiao , et al. 2004. “Arabidopsis CYP86A2 Represses *Pseudomonas syringae* Type III Genes and Is Required for Cuticle Development.” EMBO Journal 23, no. 14: 2903–2913. 15241470 10.1038/sj.emboj.7600290 PMC514950

[pbi70419-bib-0041] Xu, Y. , S. Chen , S. Zhao , et al. 2024. “Effects of Light Intensity on the Photosynthetic Characteristics of Hosta Genotypes Differing in the Glaucousness of Leaf Surface.” Scientia Horticulturae 327: 112834.

[pbi70419-bib-0042] Xue, D. , X. Zhang , X. Lu , G. Chen , and Z.‐H. Chen . 2017. “Molecular and Evolutionary Mechanisms of Cuticular Wax for Plant Drought Tolerance.” Frontiers in Plant Science 8: 621. 28503179 10.3389/fpls.2017.00621 PMC5408081

[pbi70419-bib-0043] Yang, H. , W. Mei , H. Wan , R. Xu , and Y. Cheng . 2021. “Comprehensive Analysis of KCS Gene Family in Citrinae Reveals the Involvement of CsKCS2 and CsKCS11 in Fruit Cuticular Wax Synthesis at Ripening.” Plant Science 310: 110972. 34315590 10.1016/j.plantsci.2021.110972

[pbi70419-bib-0044] Yang, J. , M. I. Ordiz , J. G. Jaworski , and R. N. Beachy . 2011. “Induced Accumulation of Cuticular Waxes Enhances Drought Tolerance in Arabidopsis by Changes in Development of Stomata.” Plant Physiology and Biochemistry 49, no. 12: 1448–1455. 22078383 10.1016/j.plaphy.2011.09.006

[pbi70419-bib-0045] Yang, K. , C.‐Y. Li , J.‐P. An , et al. 2021. “The C2H2‐Type Zinc Finger Transcription Factor MdZAT10 Negatively Regulates Drought Tolerance in Apple.” Plant Physiology and Biochemistry 167: 390–399. 34404010 10.1016/j.plaphy.2021.08.014

[pbi70419-bib-0046] Yang, Y. , C. X. Cai , Y. P. Wang , Y. R. Wang , H. L. Ju , and X. H. Chen . 2023. “Cucumber Glossy Fruit 1 (CsGLF1) Encodes the Zinc Finger Protein 6 That Regulates Fruit Glossiness by Enhancing Cuticular Wax Biosynthesis.” Horticulture Research 10, no. 1: uhac237. 36643740 10.1093/hr/uhac237 PMC9832831

[pbi70419-bib-0047] Yang, Y. , Y. Zhao , W. Zheng , et al. 2022. “Phosphatidylinositol 3‐Phosphate Regulates SCAB1‐Mediated F‐Actin Reorganization During Stomatal Closure in Arabidopsis.” Plant Cell 34, no. 1: 477–494. 34850207 10.1093/plcell/koab264 PMC8773959

[pbi70419-bib-0048] Yu, X. , L. Wang , Y. Xie , et al. 2024. “OsBBP1, a Newly Identified Protein Containing DUF630 and DUF632 Domains Confers Drought Tolerance in Rice.” Plant Science 345: 112119. 38759757 10.1016/j.plantsci.2024.112119

[pbi70419-bib-0049] Zhai, X. L. , H. Y. Wu , Y. R. Wang , et al. 2022. “The Fruit Glossiness Locus, Dull Fruit (D), Encodes a C2H2‐Type Zinc Finger Transcription Factor, CsDULL, in Cucumber ( *Cucumis sativus* L.).” Horticulture Research 9, no. 1: uhac146. 36072836 10.1093/hr/uhac146 PMC9437717

[pbi70419-bib-0050] Zhang, D. , H. Yang , X. Wang , et al. 2020. “Cytochrome P450 Family Member CYP96B5 Hydroxylates Alkanes to Primary Alcohols and Is Involved in Rice Leaf Cuticular Wax Synthesis.” New Phytologist 225, no. 5: 2094–2107. 31618451 10.1111/nph.16267

[pbi70419-bib-0051] Zhang, J.‐Y. , C. D. Broeckling , L. W. Sumner , and Z.‐Y. Wang . 2007. “Heterologous Expression of Two *Medicago truncatula* Putative ERF Transcription Factor Genes, WXP1 and WXP2, in Arabidopsis Led to Increased Leaf Wax Accumulation and Improved Drought Tolerance, but Differential Response in Freezing Tolerance.” Plant Molecular Biology 64: 265–278. 17347795 10.1007/s11103-007-9150-2

[pbi70419-bib-0052] Zhang, N. , C.‐Q. Wei , D.‐J. Xu , et al. 2024. “Photoregulatory Protein Kinases Fine‐Tune Plant Photomorphogenesis by Directing a Bifunctional Phospho‐Code on HY5 in Arabidopsis.” Developmental Cell 59: 1737–1749.e7. 38677285 10.1016/j.devcel.2024.04.007

[pbi70419-bib-0053] Zhang, Y.‐L. , C.‐L. Zhang , G.‐L. Wang , et al. 2019. “Apple AP2/EREBP Transcription Factor MdSHINE2 Confers Drought Resistance by Regulating Wax Biosynthesis.” Planta 249: 1627–1643. 30826884 10.1007/s00425-019-03115-4

[pbi70419-bib-0054] Zhao, J.‐J. , X. Xiang , P. Yang , et al. 2024. “Genome‐Wide Analysis of C2H2. 2 Gene Family in Populus Trichocarpa and the Function Exploration of PtrC2H2. 2–6 in Osmotic Stress.” International Journal of Biological Macromolecules 283: 137937. 39579826 10.1016/j.ijbiomac.2024.137937

[pbi70419-bib-0055] Zhao, P. , Q. Li , Y. Lei , J. Zou , and Q. Li . 2025. “Adaptation of Cuticle Metabolism to Abiotic Stress in Plants.” Crop and Environment 4, no. 1: 38–44.

[pbi70419-bib-0056] Zhao, Y. , Q. Liu , X. Wang , et al. 2024. “ZmCER1, a Putative ECERIFERUM 1 Protein in Maize, Functions in Cuticular Wax Biosynthesis and Bulliform Cell Development.” Crop Journal 12: 743–752.

