# Supplementary material for: The PtrC2H2.2‐6‐PtrCYP86A7/A8 Module Regulates Poplar Drought Tolerance Through Mediating Cutin and Wax Biosynthesis Pathways
Source: Plant Biotechnol J. 2025 Oct 24;24(3):1428–45. doi: 10.1111/pbi.70419 (PMC12946459; doi:10.1111/pbi.70419)
Supplement: Supplementary file 1 — Figure S1: GCMS‐related data. Figure S2: Identifying PtrCYP86A7‐overexpressing transgenic poplars. Figure S3: Identifying PtrCYP86A8‐overexpressing transgenic poplars. Figure S4: Morphological differences between Ox‐PtrCYP86A7, Ox‐PtrCYP86A8 and the wild type in short‐term drought assays. Figure S5: Y2H screening procedure using PtrC2H2.2‐6 as a bait protein. Figure S6: pbi70419‐sup‐0001‐Supinfo.docx. PtrPPK1 is localised in the nucleus. Figure S7: Tissue expression pattern of PtrCYP86A7/A8 in P. trichocarpa . Figure S8: Absolute expression level (TPM for RNA‐seq) of genes related to cutin and wax synthesis in the PtrC2H2.2‐6 overexpression poplar vs. WT group. Figure S9: Relative expression of PtrPPK1 following drought and ABA treatment. Figure S10: The expression of PtrPPK1 in different plants. Figure S11: Phenotypic characterisation and statistics of different lines under prolonged drought conditions. Figure S12: Co‐expression network diagram of genes involved in the wax, suberin and cutin synthesis pathway with PtrC2H2.2‐6 and other transcription factors. Figure S13: PtrPPK1 phosphorylates PtrC2H2.2‐6 in vivo. Figure S14: Overexpression of PtrPPK1 in poplar enhances plant resistance to drought stress. Figure S15: The relative expression level of genes related to ROS and ABA signal transduction in the PtrPPK1 overexpression poplar vs. WT group. Methods S1: Plant growth conditions and treatments. Methods S2: RNA extraction and RT‐qPCR. Methods S3: Subcellular localization. Methods S4: Y2H‐seq, self‐activation detection and Y2H assay. Methods S5: Drought and mannitol treatments. Methods S6: Measurement and analysis of physiological indicators. Methods S7: DAB and NBT staining. Methods S8: Thermal Imaging. Methods S9: Analysis of chlorophyll fluorescence. Methods S10: Leaf pre‐processing for SEM photography. Methods S11: Observation of leaf longitudinal section staining. Methods S12: Leaf pre‐processing for GC–MS. Methods S13: Transcriptome analysis. Methods S14: Ex [file PBI-24-1428-s002.docx]

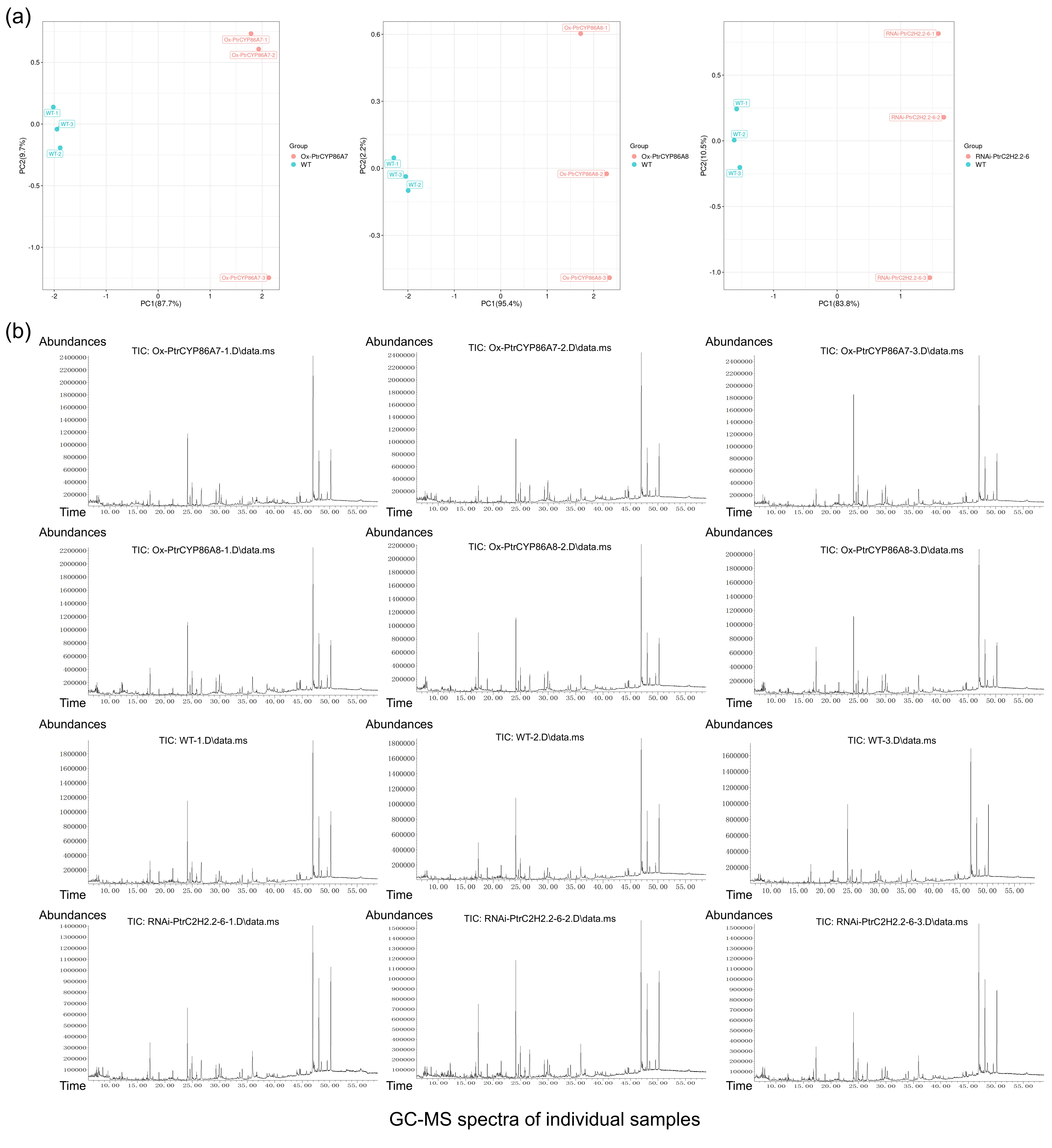


**Figure S1.** GCMS-related data. (a) PCA analysis between different transgenic lines and WT. The horizontal axis represents the contribution of principal component 1 (PC1) to the differentiated sample, and the vertical axis represents the contribution of principal component 2 (PC2) to the differentiated sample. (b) GC-MS spectrum of the samples. Horizontal coordinates represent time, and vertical coordinates represent abundance.


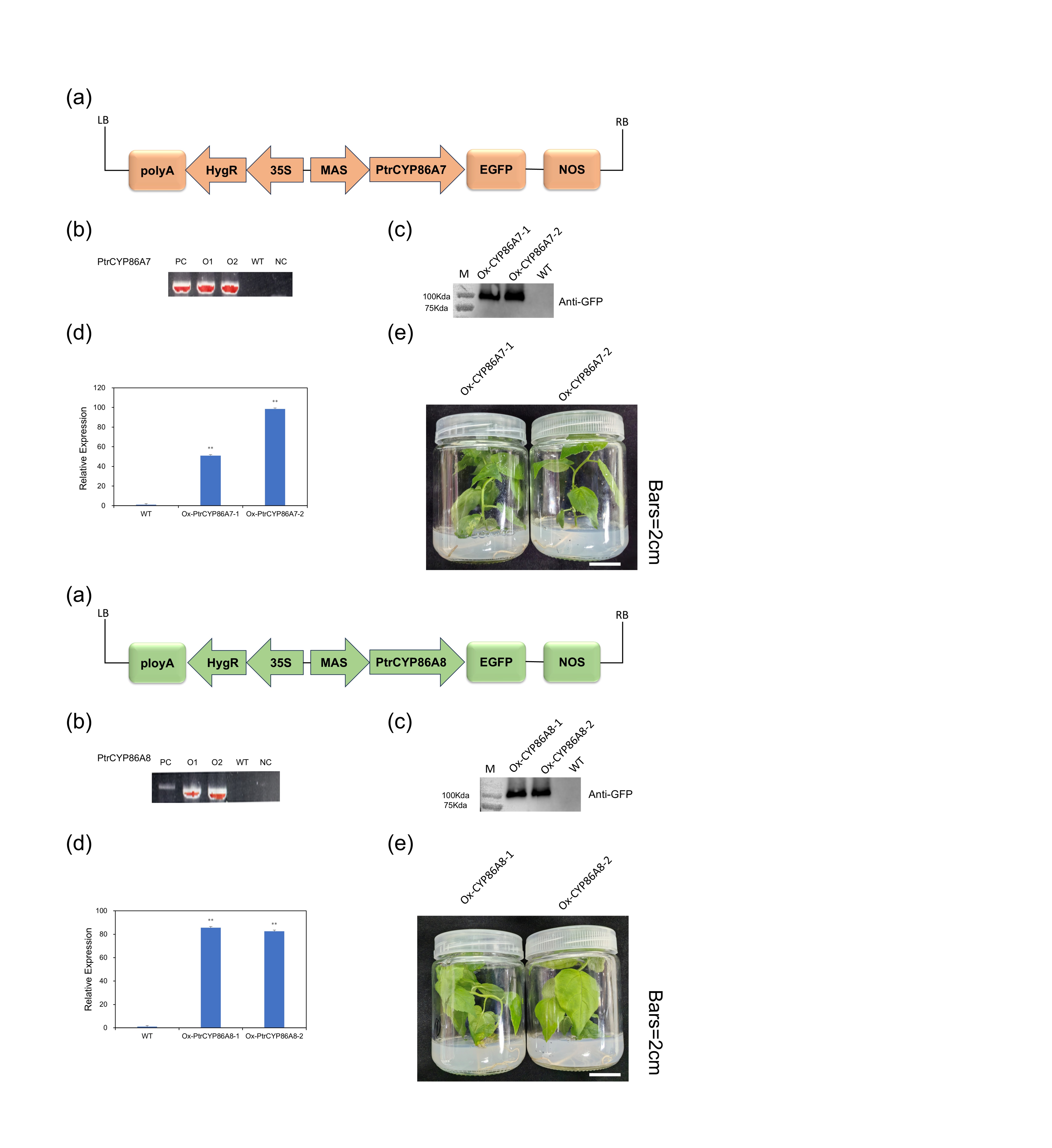


**Figure S2.** Identifying *PtrCYP86A7*-overexpressing transgenic poplars. (a) Schematic representation of the structure of overexpressing *PtrCYP86A7*. (b) PCR identification of transgenic plants. PC, positive control, O-*PtrCYP86A7* plasmid DNA as positive control; NC, negative control, double-distilled water as negative control; WT: wild type; O1-O2: overexpressing plants. (c) The expression of the PtrCYP86A7 protein in different transgenic lines was examined using the western blot technique. M: Marker. The predicted size of the PtrCYP86A7-GFP protein is approximately 90 kDa. (d) Real-time quantitative PCR analysis of *PtrCYP86A7* expression levels in different transgenic lines. Values are means ± SD (n = 9). Asterisks denote significant differences: **, P ≤ 0.01. (e) Two overexpressing *PtrCYP86A7* transgenic lines.


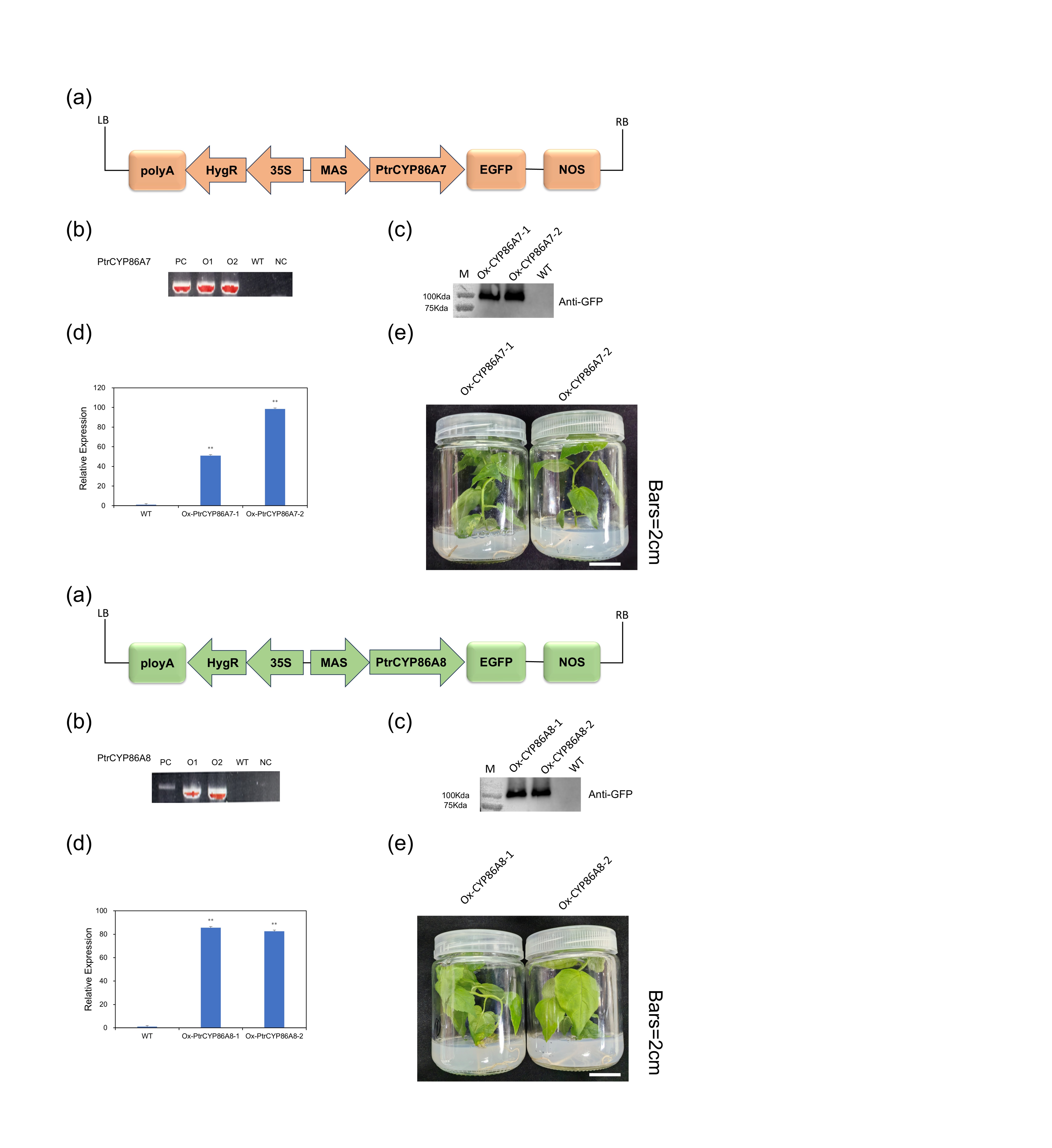


**Figure S3.** Identifying *PtrCYP86A8*-overexpressing transgenic poplars. (a) Schematic representation of the structure of overexpressing *PtrCYP86A8*. (b) PCR identification of transgenic plants. PC, positive control, Super-*PtrCYP86A8* plasmid DNA as positive control; NC, negative control, double-distilled water as negative control; WT: wild type; O1-O2: overexpressing plants. (c) The expression of the PtrCYP86A8 protein in different transgenic lines was examined using the western blot technique. M: Marker. The predicted size of the PtrCYP86A8-GFP protein is approximately 90 kDa. (d) Real-time quantitative PCR analysis of *PtrCYP86A8* expression levels in different transgenic lines. Values are means ± SD (n = 9). Asterisks denote significant differences: **, P ≤ 0.01. (e) Two overexpressing *PtrCYP86A8* transgenic lines.


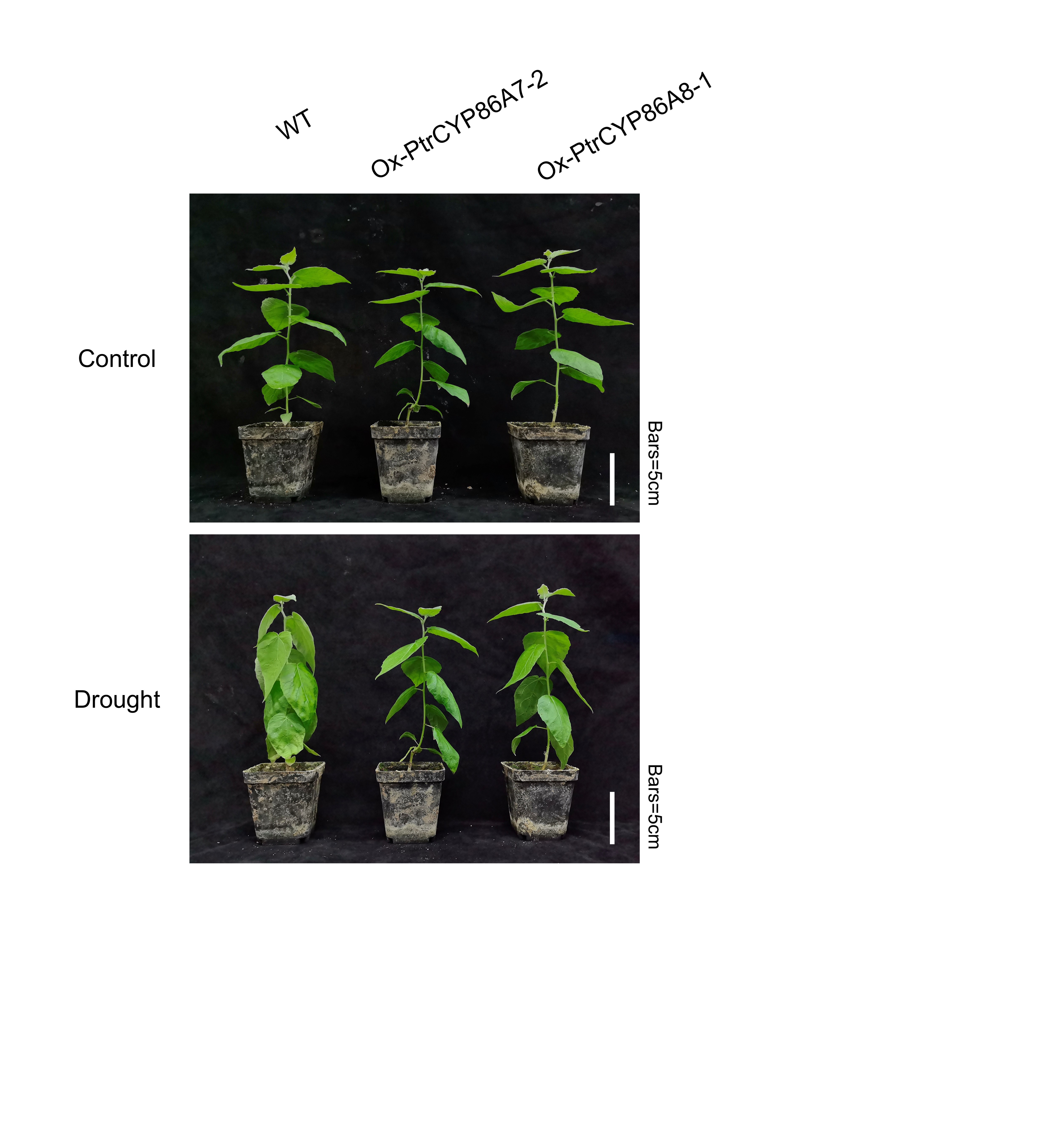


**Figure S4.** Morphological differences between Ox-*PtrCYP86A7*-2, Ox-*PtrCYP86A8*-*1*, and the wild type in short-term drought assays. Bar = 5 cm.


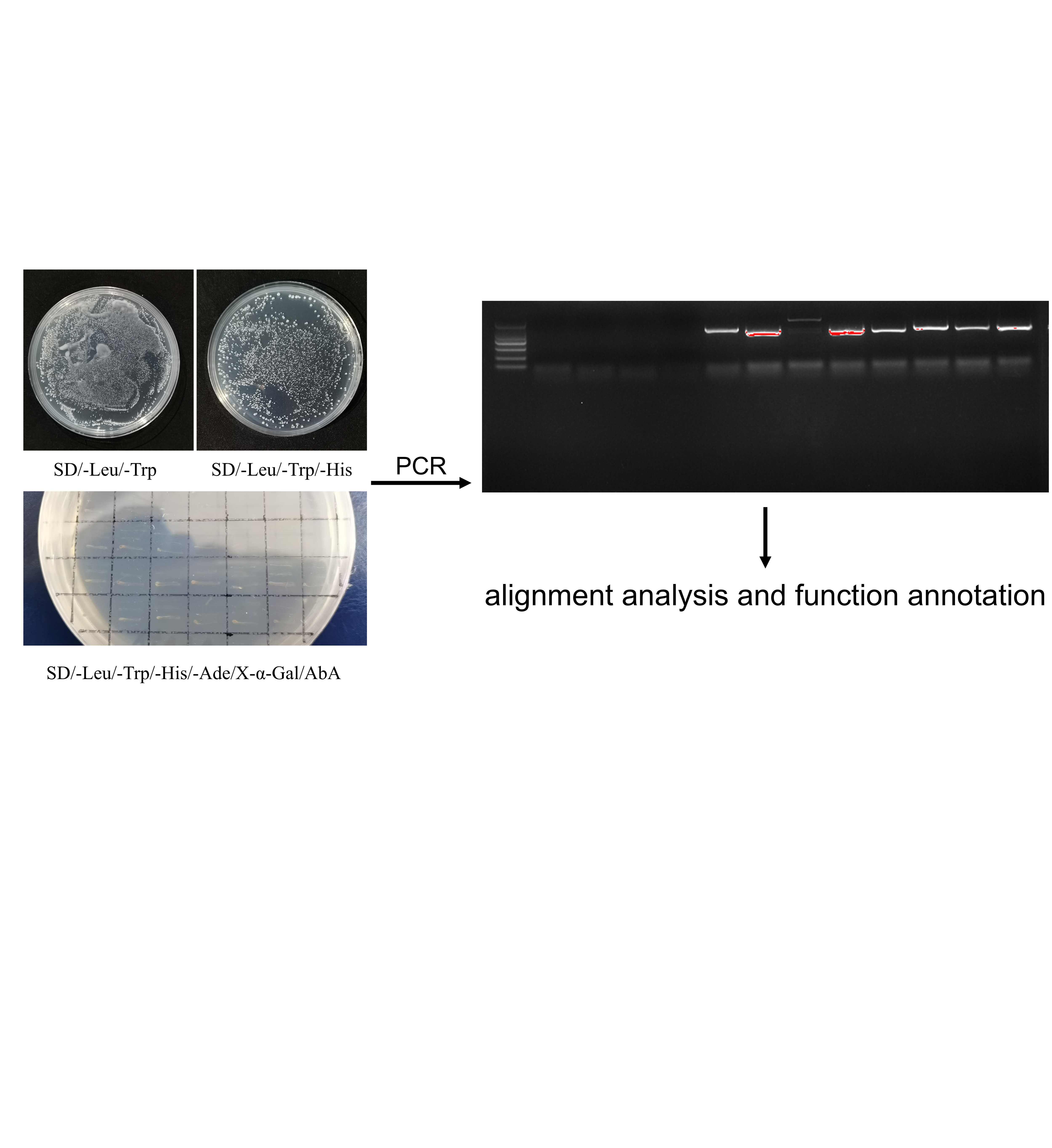


**Figure S5.** Y2H screening procedure using P*trC2H2.2-6* as a bait protein. Yeast were grown on SD/-Leu/-Trp, SD/-Leu/-Trp/-His, SD/-Leu/-Trp/-His/-Ade/X-α-Gal/AbA medium, and finally picked blue single colonies on SD/-Leu/-Trp/-His/-Ade/X-α-Gal/AbA for PCR detection and sequenced, and the sequencing results are available on the phytozome website for sequence comparison.


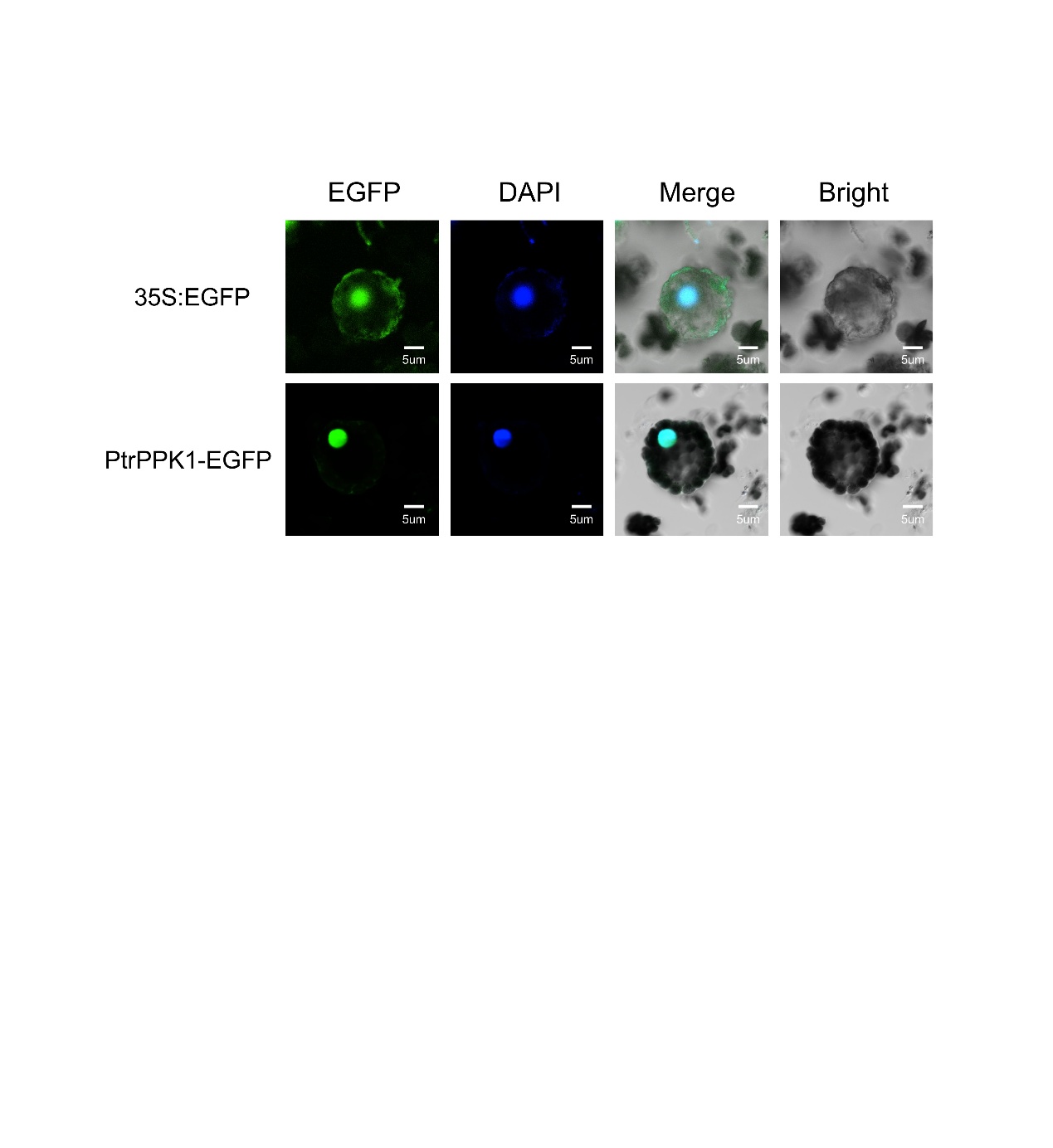


**Figure S6.** *PtrPPK1* is localized in the nucleus. *PtrPPK1*-EGFP in transiently expressed tobacco leaf protoplasts. DAPI (blue) was applied to mark the nucleus. Bars = 5 μm.


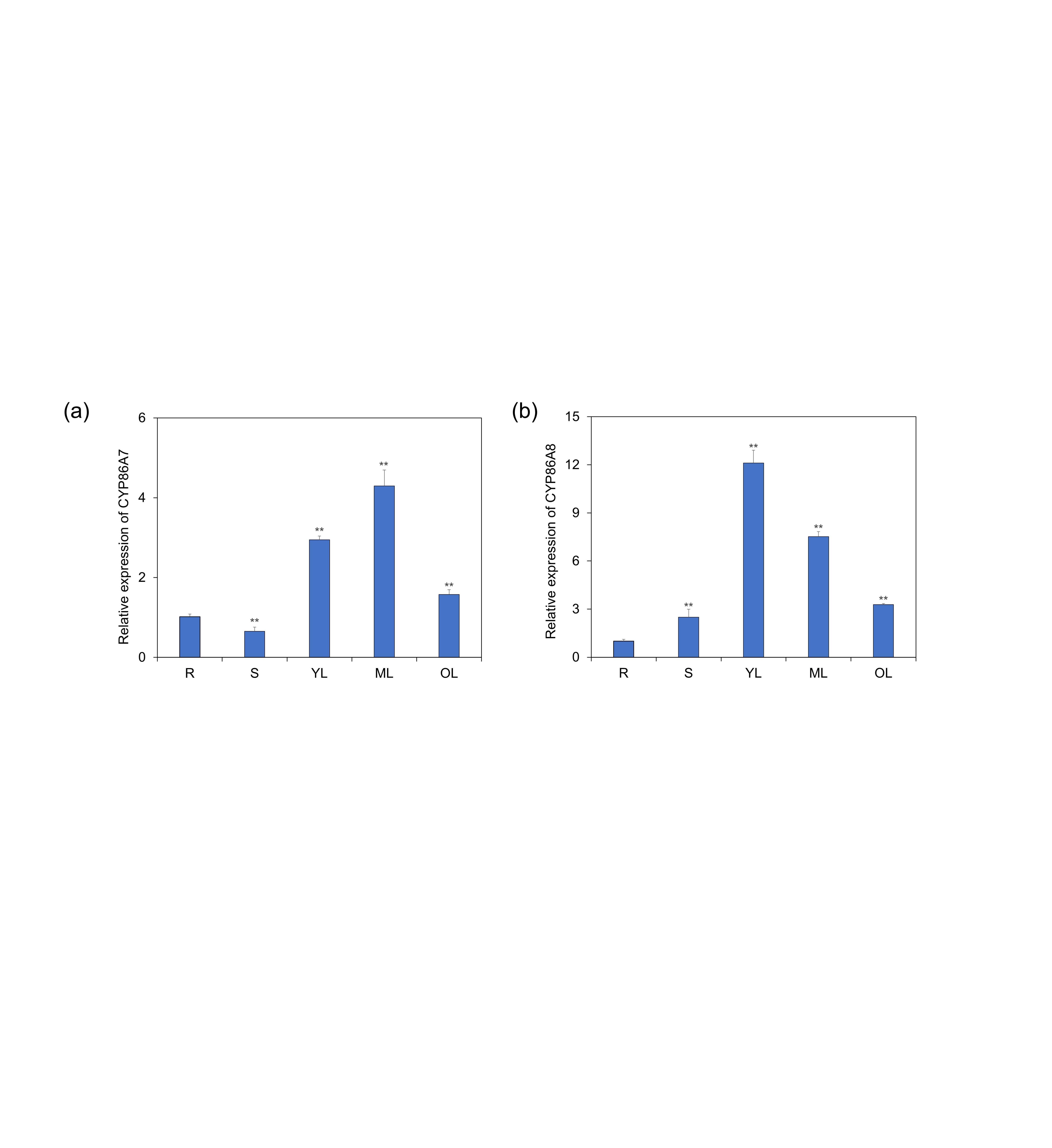


**Figure S7.** Tissue expression pattern of *PtrCYP86A7/A8* in *P. trichocarpa*. (a) The transcript levels of *PtrCYP86A7* in different tissues of *P. trichocarpa*. R: root; S: stem; YL: young leaf; ML: mature leaf; OL: old leaf. Values are means ± SD (n = 9). Asterisks denote significant differences. **, P ≤ 0.01. (b) The transcript levels of *PtrCYP86A8* in different tissues of *P. trichocarpa*. R: root; S: stem; YL: young leaf; ML: mature leaf; OL: old leaf. Values are means ± SD (n = 9). Asterisks denote significant differences. **, P ≤ 0.01.


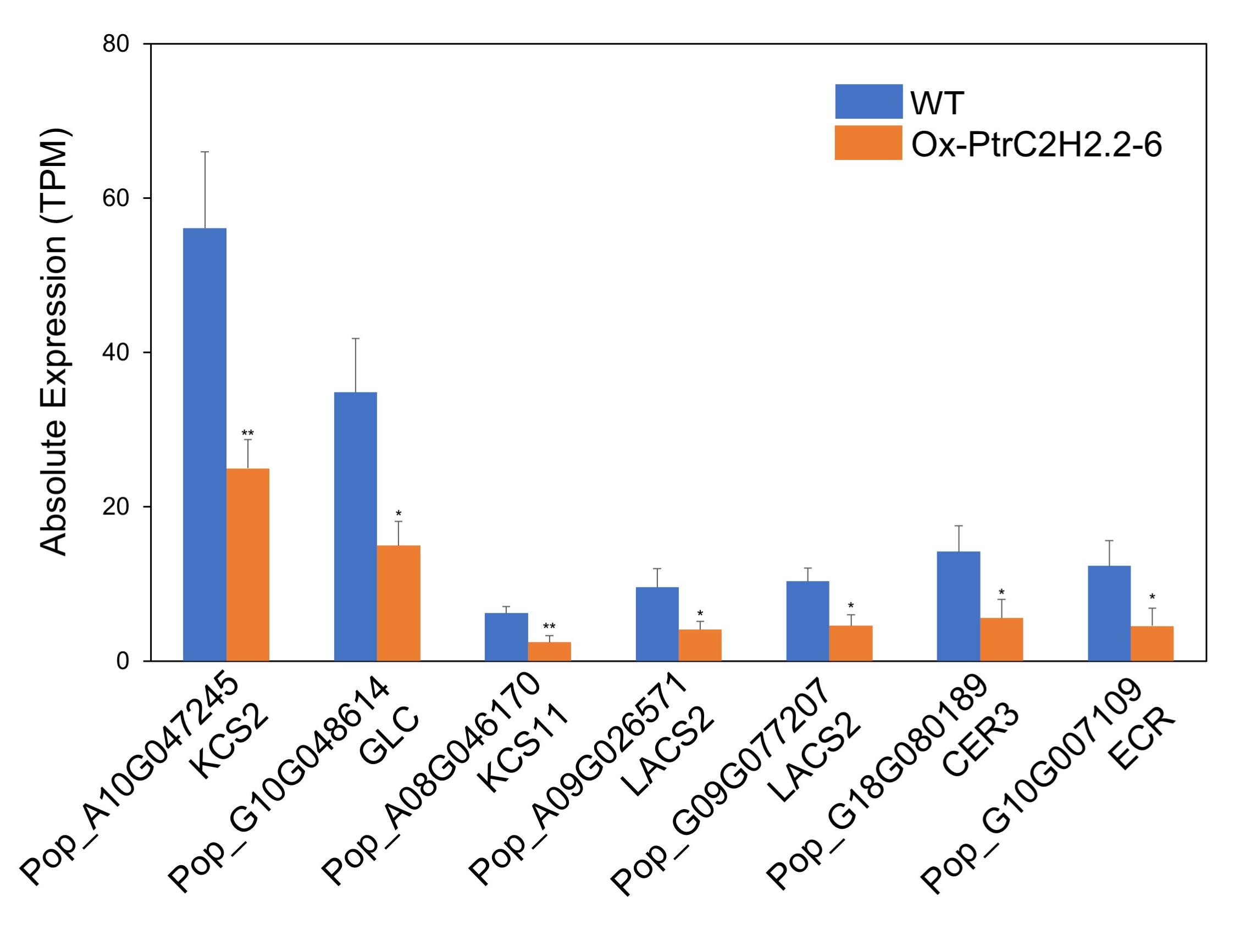


**Figure S8.** Absolute expression level (TPM for RNA-seq) of genes related to cutin and wax synthesis in the *PtrC2H2.2-6* overexpression poplar vs WT group. Blue represents wild-type plants, orange represents Ox-*PtrC2H2.2-6* plants. Values are means ± SD (n =3). Asterisks denote significant differences. *, P ≤ 0.05; **, P ≤ 0.01


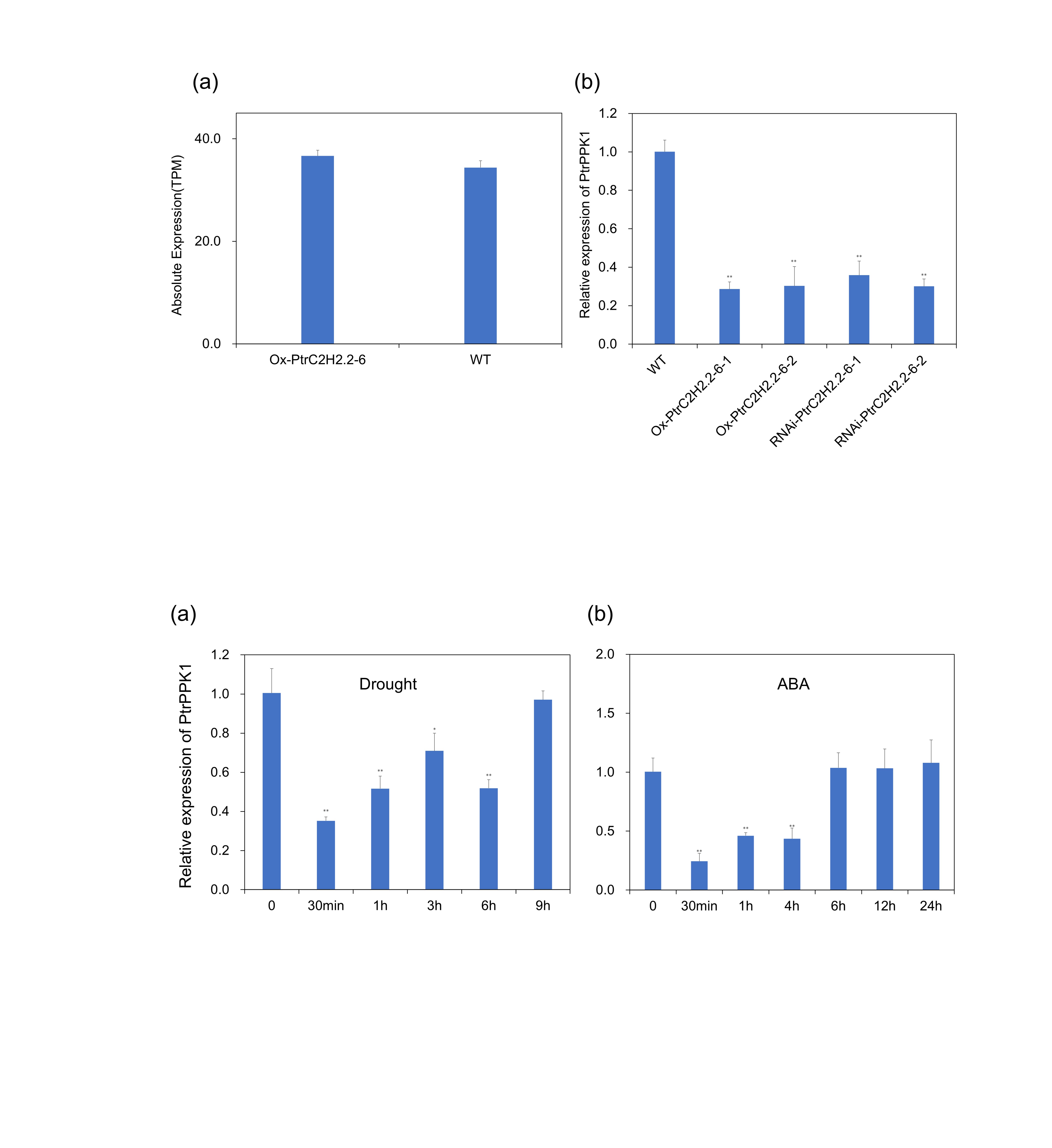


**Figure S9.** Relative expression of *PtrPPK1* following drought and ABA treatment. (a) The transcript levels of *PtrPPK1* in leaf tissues were monitored at different times of dehydration. Values are means ± SD (n = 9). Asterisks denote significant differences. ∗, P ≤ 0.05; ∗∗, P ≤ 0.01. (b) The transcript levels of *PtrPPK1* in leaf tissues were evaluated at various time points following ABA treatment. Values are means ± SD (n = 9). Asterisks denote significant differences. ∗∗, P ≤ 0.01.


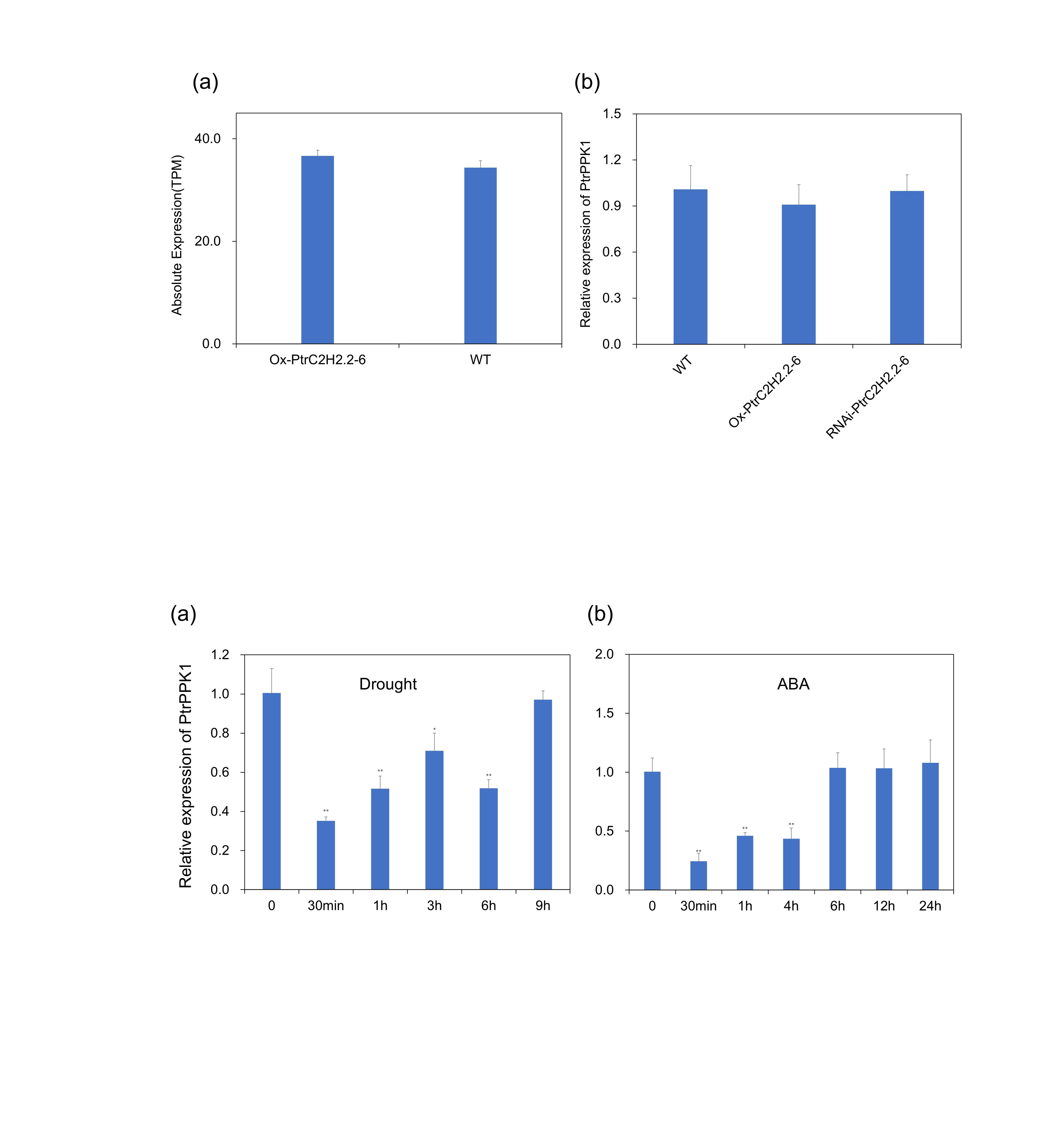


**Figure S10.** The expression of *PtrPPK1* in different plants. (a) Absolute expression level (TPM for RNA-seq) of *PtrPPK1* in the *PtrC2H2.2-6* overexpression poplar vs WT group. Values are means ± SD (n = 3). (b) The transcript levels of *PtrPPK1* in leaves of 4-week-old WT, Ox-*PtrC2H2.2-6*, and RNAi-*PtrC2H2.2-6* plants. Values are means ± SD (n = 9).


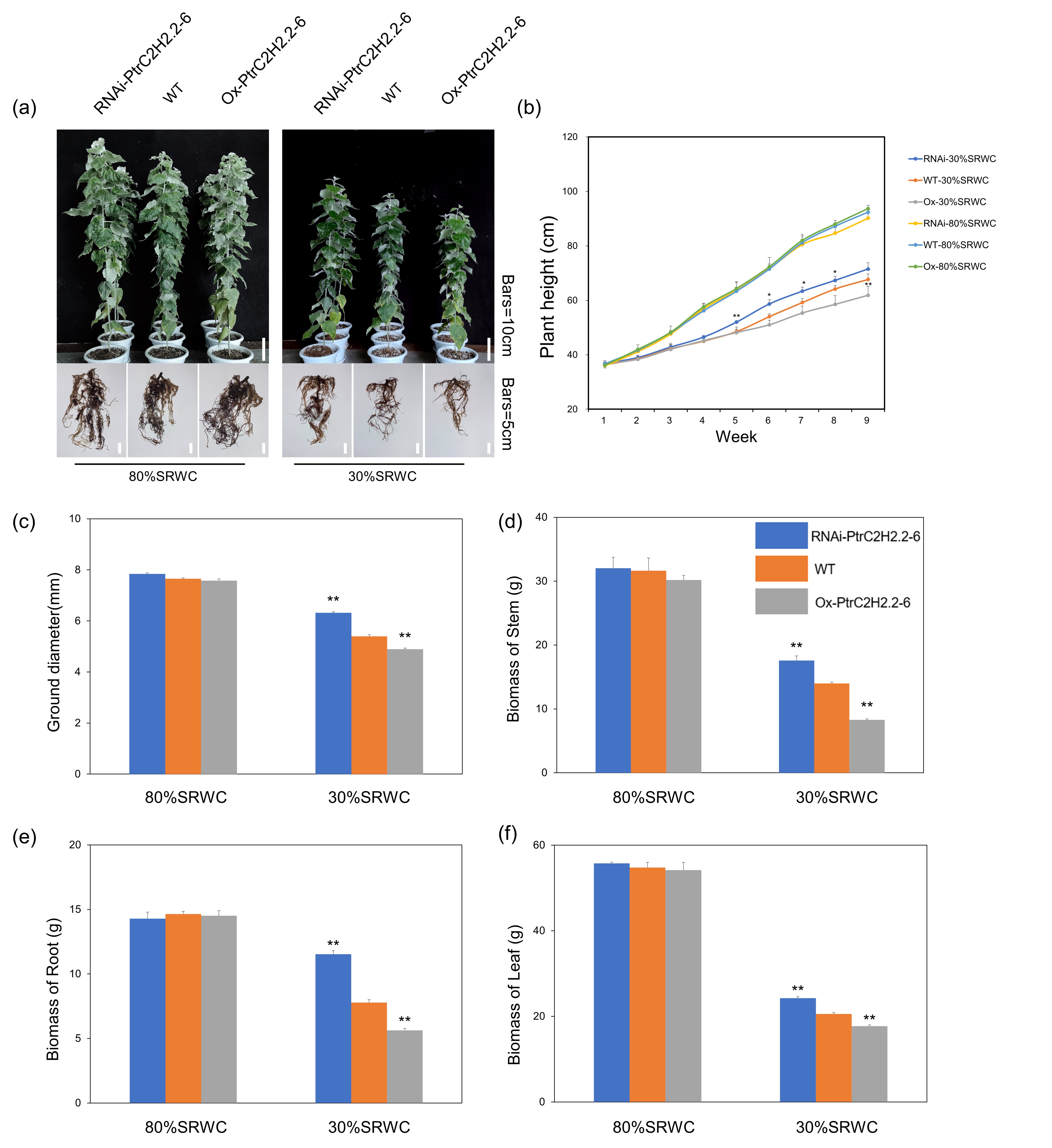


**Figure S11.** Phenotypic characterization and statistics of different lines under prolonged drought conditions. (a) Phenotypes, root length plots of WT, Ox-PtrC2H2.2-6 and RNAi-PtrC2H2.2-6 plants after 80% SRWC and 30% SRWC treatments, respectively. (b) Plant height statistics of WT, Ox-PtrC2H2.2-6 and RNAi-PtrC2H2.2-6 plants under 80% SRWC and 30% SRWC treatments, respectively. (c) Ground diameter statistics of WT, Ox-PtrC2H2.2-6 and RNAi-PtrC2H2.2-6 plants under 80% SRWC and 30% SRWC treatments, respectively. (d) Stem biomass fresh weight statistics of WT, Ox-PtrC2H2.2-6 and RNAi-PtrC2H2.2-6 plants after 80% SRWC and 30% SRWC treatments, respectively. (e) Root biomass fresh weight statistics of WT, Ox-PtrC2H2.2-6 and RNAi-PtrC2H2.2-6 plants after 80% SRWC and 30% SRWC treatments, respectively. (f) Leaf biomass fresh weight statistics of WT, Ox-PtrC2H2.2-6 and RNAi-PtrC2H2.2-6 plants after 80% SRWC and 30% SRWC treatments, respectively. Values are means ± SD (n = 30) and asterisks indicate significant differences: *, P ≤ 0.05; **, P ≤ 0.01.


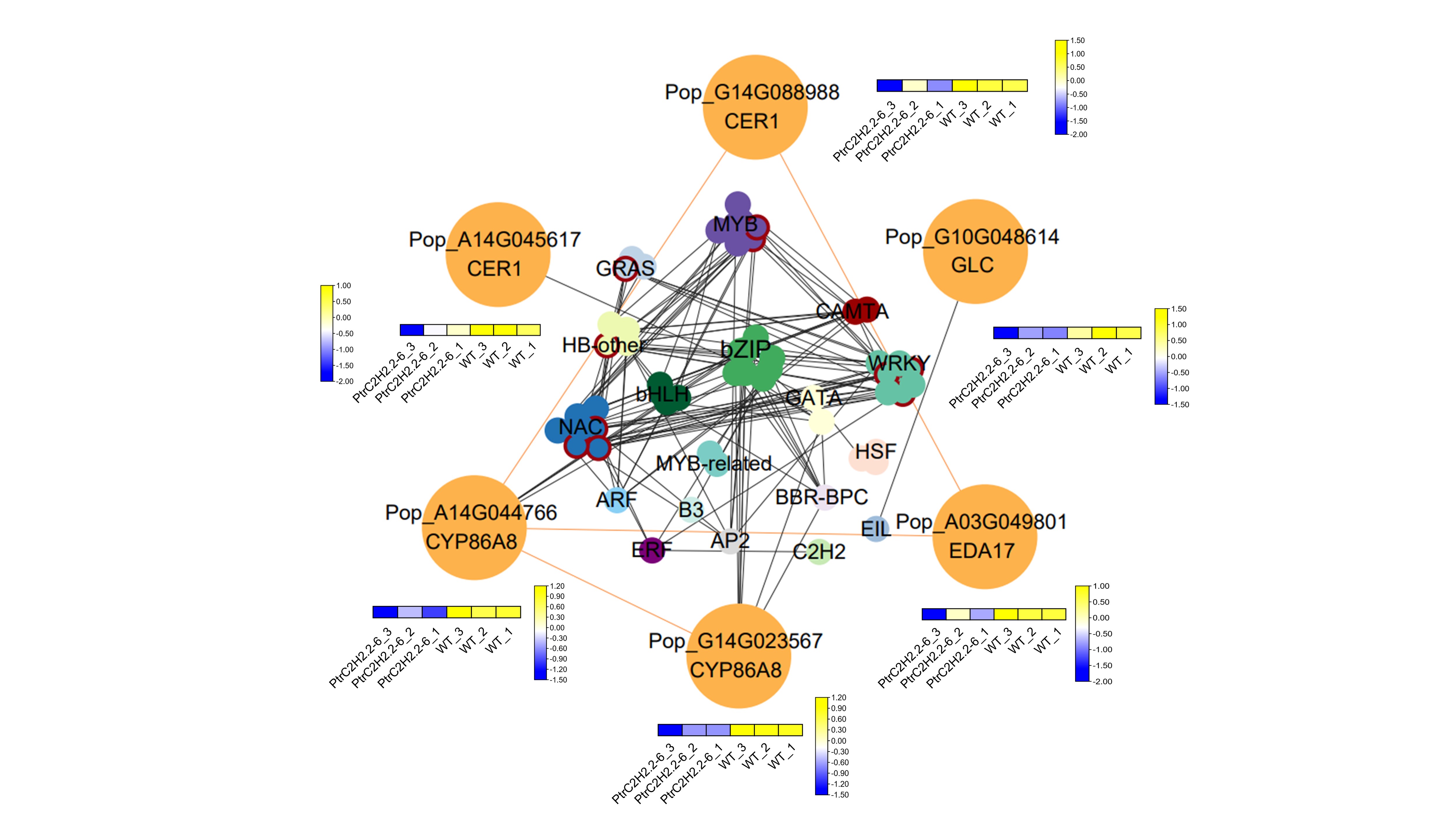


**Figure S12.** Co-expression network diagram of genes involved in the wax, suberin, and cutin synthesis pathway with *PtrC2H2.2-6* and other transcription factors. Different colours represent different types of transcription factors.


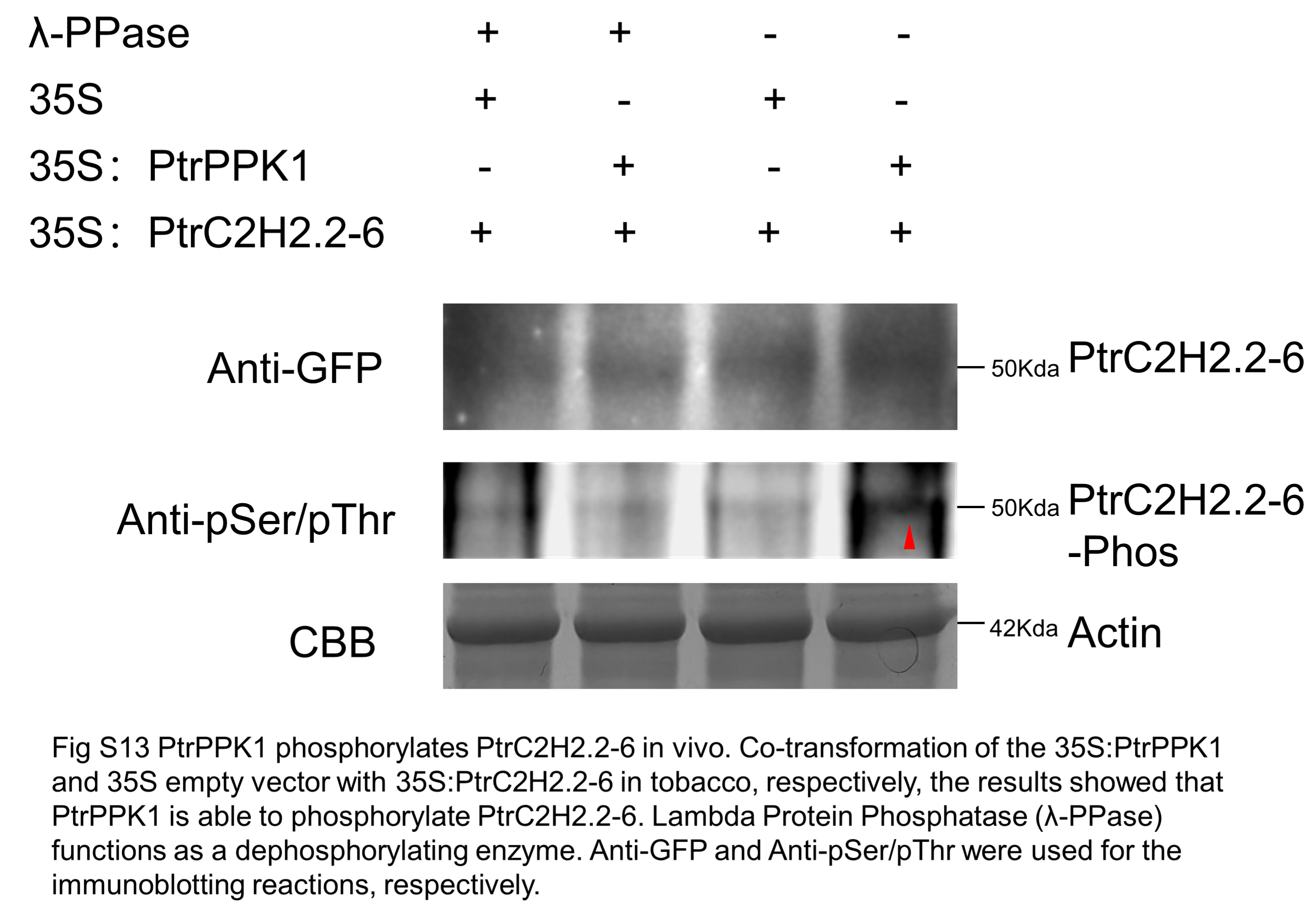
**Figure S13.** PtrPPK1 phosphorylates PtrC2H2.2-6 in vivo. Co-transformation of the 35S:PtrPPK1 and 35S empty vector with 35S:PtrC2H2.2-6 in tobacco, respectively, the results showed that PtrPPK1 is able to phosphorylate PtrC2H2.2-6. Lambda Protein Phosphatase (λ-PPase) functions as a dephosphorylating enzyme. Anti-GFP and Anti-pSer/pThr were used for the immunoblotting reactions, respectively.


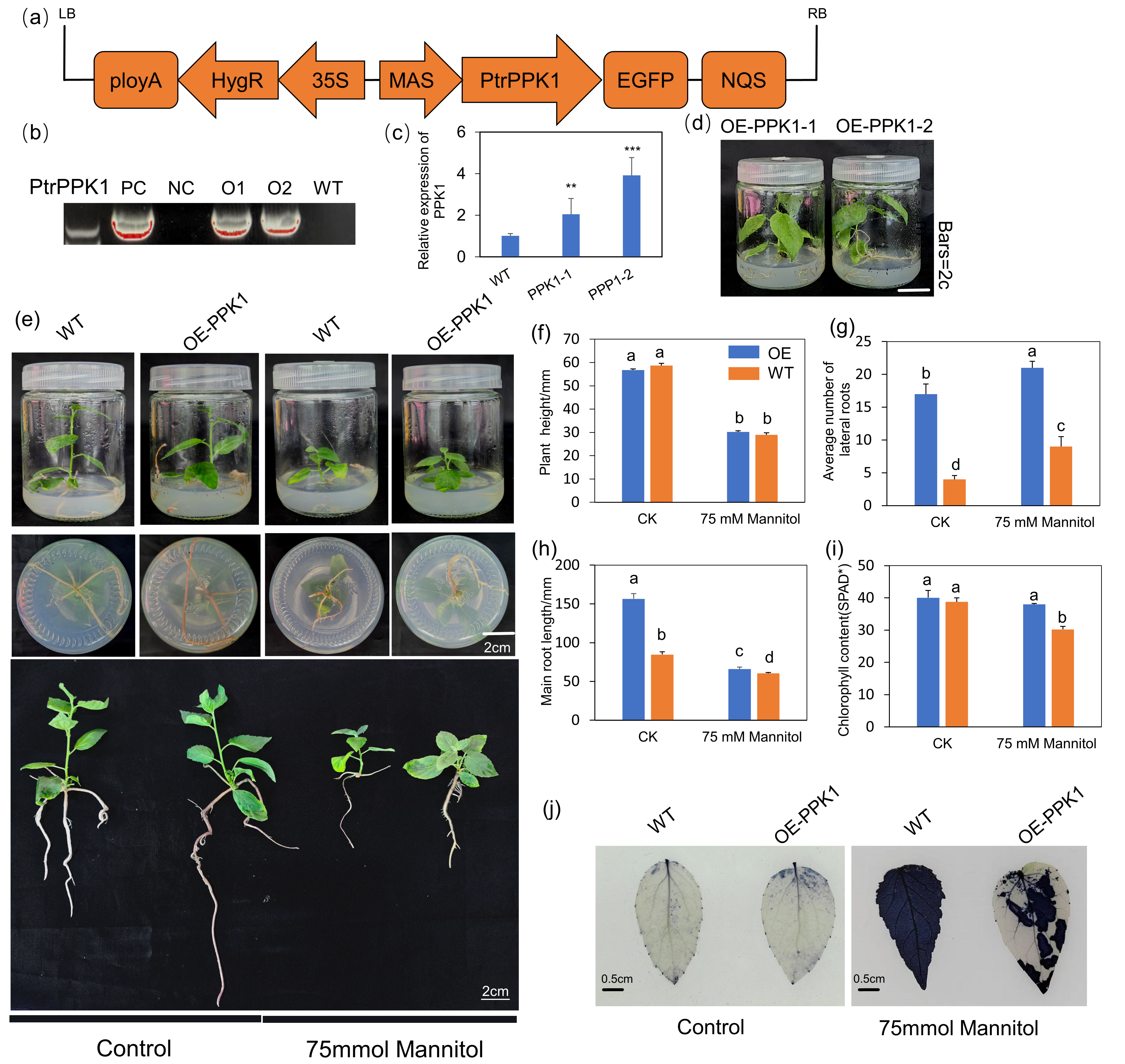


**Figure S14.** Overexpression of PtrPPK1 in Poplar Enhances Plant Resistance to Drought Stress. (a) Schematic representation of the structure of overexpressing *PtrPPK1*. (b) PCR identification of transgenic plants. PC, positive control, Super-*PtrPPK1* plasmid DNA as positive control; NC, negative control, double-distilled water as negative control; WT: wild type; O1-O2: overexpressing plants. (c) Real-time quantitative PCR analysis of *PtrPPK1* expression levels in different transgenic lines. Values are means ± SD (n = 9). Asterisks denote significant differences: **, P ≤ 0.01, ***, P ≤ 0.001. (d) Two overexpressing *PtrPPK1* transgenic lines. (e) Phenotypic comparison of PPK1-overexpressing (OE-PPK1) and wild-type (WT) poplar seedlings grown for 27 d on normal medium or on medium supplemented with 75 mM mannitol (simulating drought stress using mannitol); bar = 2 cm. (f–i) Plant height (f), average number of lateral roots (g), main root length (h), and chlorophyll content (i) of OE-PPK1 and WT under control and mannitol-stress conditions. Data are means ± SD of three independent experiments. Different letters (a, b, c, d) indicate significant differences (P < 0.05). (j) NBT staining of O_₂_^⁻^ accumulation in leaves of OX-PPK1 and WT seedlings after 75 mM mannitol treatment; bars = 0.5 cm.


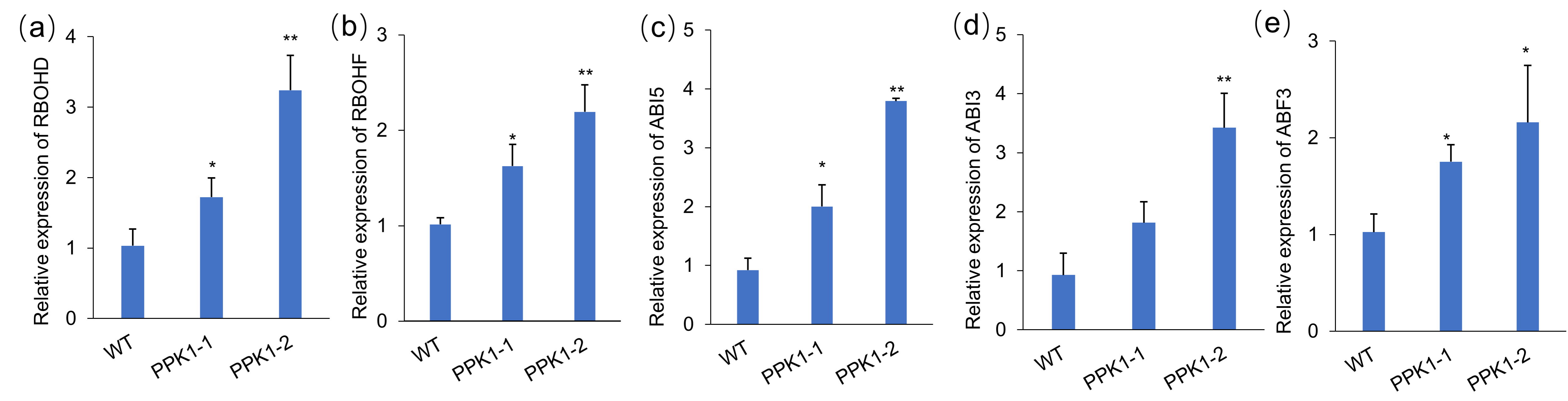


**Figure S15.** The relative expression level of genes related to ROS and ABA signal transduction in the *PtrPPK1* overexpression poplar vs WT group. (a-b) Relative expression levels of ROS signaling-related genes (RBOHD/F) in different plants. (c-e) Relative expression levels of ABA signaling-related genes (ABI5/3 and ABF3) in different plants. Values are means ± SD (n =4). Asterisks denote significant differences. *, P ≤ 0.05; **, P ≤ 0.01

**Supplementary Methods**

*Methods S1 Plant growth conditions and treatments*

Poplar trees were grown under meticulous greenhouse settings in Wenjiang, Chengdu, China. The plants were subjected to a light regime of 16 hours of illumination and 8 hours of darkness, with the temperature maintained at a steady 25°C and the relative humidity kept at 70%. The geographical coordinates for this location are 30°70′N latitude and 103°85′E longitude, with an elevation of 537.11 meters above sea level. To explore the expression profile of the gene *PtrC2H2.2-6* across various plant parts, samples were collected from 2-month-old P. trichocarpa, encompassing young leaves (YL), mature leaves (ML), old leaves (OL), roots (R), and stems (S). For the ABA treatment, a solution of 200 µM ABA was applied to 2-month-old P. trichocarpa seedlings at intervals of 0, 0.5, 1, 2, 4, 6, 12, and 24 hours. Dehydration stress was induced by removing the plants from their soil and subjecting them to aerial exposure for durations of 0, 0.5, 1, 3, 6, and 9 hours. Each trial involved a minimum of 20 seedlings, with leaf samples being harvested at the specified time intervals, flash-frozen in liquid nitrogen, and subsequently preserved at -80°C. The specific methodology is referenced from a published article (J. Li et al., 2024).

*Methods S2 RNA extraction and RT-qPCR*

Total RNA was extracted and purified using the OminiPlant RNA Kit (DNase I) (CW2598S, CWBIO, Beijing, China) in accordance with the manufacturer's instructions. The OD260/OD280 ratio measured via a Nano Photometer N60Touch (Implen, Munich, Germany) and electrophoresis was used to assess RNA quality. Subsequently, we used the ExonScript RT SuperMix with dsDNase (A502-01, Exongen, Chengdu, China) to reverse RNA to cDNA (Meng et al., 2019). The RT-qPCR procedure was conducted in accordance with the instructions provided by the manufacturer (Tsingke, Beijing, China). The amplification phase of the PCR was conducted using a Bio-Rad CFX96 real-time PCR detection system (Bio-Rad, Hercules, CA, USA). To ascertain the relative expression levels of the genes of interest, the 2-ΔΔCt method was employed for comparative quantification (F. He et al., 2020). For each treatment point, a total of 9 replicates were included, which consisted of three biological replicates and three technical replicates. The average value was taken for the three technical replicates. A list of primers used is given in Supporting Information Table S5.

*Methods S3 Subcellular localization*

In order to know the subcellular localization of *PtrC2H2.2-6* and *PtrPPK1*, the 35S:*PtrC2H2.2-6*-EGFP and 35S:*PtPPK1*-EGFP fusion proteins were instantaneously transfected into protoplasts (Duan et al., 2017). Fluorescence signals of the samples were observed using a laser confocal microscope FV3000 (OLYMPUS, Japan). The following lines of the argon-ion laser were used: 488 nm for GFP, 380 nm for DAPI, and 488 nm for chlorophyll. Fluorescence was detected at 495-515 nm for GFP, 430-450 nm for DAPI, and 650 nm for chlorophyll.

*Methods S4 Y2H-seq, self-activation detection, and Y2H assay*

For the assessment of self-activation capabilities, we transformed yeast cells with the pGALBD-*PtrC2H2.2-6* construct and plated them on synthetic dropout (SD) medium lacking tryptophan (-Trp), leucine (-Leu), histidine (-His), and adenine (-Ade) and supplemented with X-α-galactosidase (x-a-gal). After incubation at 30°C, no obvious blue spots appeared, which indicated that *PtrC2H2.2-6* was not self-activating, and therefore further experimental procedures could be carried out.

To investigate the proteins that interact with *PtrC2H2.2-6*, a pGBKT7-*PtrC2H2.2-6* fusion vector was constructed as bait, and a yeast library was constructed as prey by OE Biotech. Co., Ltd. (Shanghai, China). The transformants of AD and BD were placed on SD-Leu-Trp plates and incubated for 2 d at 30 ℃. The interactions were tested on SD/-Leu/-Trp/-His/-Ade plates and incubated for 3-4 d at 30 ℃. All single yeast colonies were screened using SD/-Leu/-Trp/-His/-Ade media supplemented with Aureobasidin A (AbA, coolaber). Finally, multiple single colonies obtained on SD/-Leu/-Trp/-His/-Ade/X-a-Gal/ABA selective media were subjected to PCR, and P. trichocarpa v4.1 was selected as a functional annotation.

To investigate the interaction between *PtrC2H2.2-6* and *PtrPPK1*, those were cloned into pGBKT7 (BD) (Takara Bio USA, Mountain View, CA, USA) and pGADT7 (AD) (Takara Bio), respectively. For Y2H, the bait and prey plasmids were co-transformed into the Y2HGold strain (Weidi Biotechnology Co., Ltd., Shanghai, China). And yeast cells were grown on SD/-T/-L and SD/-L/-T/-H/-A medium for 5 d, respectively, and photographed. pGBKT7-53 and pGADT7-T were used as positive controls, and pGBKT7-Lam and pGADT7-T were used as negative controls.

*Methods S5 Drought and mannitol treatments*

For short-term drought treatment, 2-month-old transgenic and WT poplars with consistent growth conditions were screened for a short-term drought treatment in the greenhouse. All plants were grown in appropriately sized pots (12 cm x 12 cm x 12 cm) with a tray at the bottom of each pot. For the drought group, the soil RWC values decreased from 70%, whereas those of the control group remained at 70%, and all other experimental conditions were the same (F. He et al., 2018).

For mannitol treatment, the terminal buds of Ox-*PtrCYP86A7*, Ox-*PtrCYP86A8*, and WT sterile seedlings with uniform growth conditions and approximately the same growth height were transferred into WPM medium only containing 100 mM mannitol for one month to simulate osmotic stress (Tong et al., 2021). The control was WPM medium without any other additions. Mannitol is for modeling osmotic stress. Harvested after one month of growth and photographed to record phenotypes.

Leaves from the same sites were harvested after treatment for subsequent determination of physiological indices, including MDA content, relative water content, relative electrical conductance, and DAB and NBT staining as described for Methods S6-7. In addition, thermographic photographs were taken of the leaves of wild-type and transgenic plants in the drought-treated group as well as in the control group, and their chlorophyll fluorescence parameters were determined, as shown in Methods S8-9.

*Methods S6 Measurement and analysis of physiological indicators*

For the determination of MDA and REC, leaf material from control and drought-treated transgenic lines and wild type was measured according to previously published protocols (Shi et al., 2013, 2014). MDA (mol/g FW) = (6.45 * (A532 - A600) - 0.56 * A450) * 9Vt5 - 1FW - 1 [Vt: total volume of extract (mL); Vs: extract volume measured with (mL) to determine the extract volume]. A DDSJ-319L conductivity meter (Leici-DDSJ-319L, Shanghai, China) was used to detect the conductivity of the supernatant (L1). These experiments were independently repeated five times under the same conditions.

The 10th–13th leaves of control- and drought-treated transgenic lines and wild type were selected for RWC measurements. Briefly, we determined leaf FW (removed fresh leaves weighed), leaf swelling weight (TW, measured after 12 h of immersion in water), and leaf dry weight (DW, measured after 72 h of drying at 80 °C). RWC was calculated as (FW - DW)/(TW - DW) x 100%.

*Methods S7 DAB and NBT staining*

Leaves of wild-type and transgenic plants in drought-treated and control groups were selected and soaked in 0.1 mg/mL diaminobenzidine (DAB) solution (Beyotime, ST033) and nitroblue tetrazolium (NBT) solution (Biosharp, BS120) for 12 h. The temperature was controlled at 25 °C, and the leaf tissues were observed in darkness for the O_2_^-^ and H_2_O_2_ accumulation levels in the leaf tissues (Ding et al., 2015; J. Liu et al., 2016). After staining, the treated and control leaves were soaked in pure alcohol for another 12 h to remove chlorophyll. Each treatment contained at least 3 biological replicates.

*Methods S8 Thermal Imaging*

For infrared thermography assays, plants were photographed using an infrared thermographer (WIRIS Pro; Height Technologies, Geldermalsen, Netherlands) after 7 days of drought treatment or control conditions (F. He et al., 2024). The data were also visualized using Excel.

*Methods S9* *Analysis of chlorophyll fluorescence*

After a 30-min dark adaptation process, we monitored photosynthesis in the 10th to 13th leaves of wild-type and transgenic plants in drought-treated and control groups using a modulated chlorophyll fluorescence imaging system (ZEAL-IMAG). With this monitoring, we aimed to determine the key parameters of Fv/Fm, Y(II), qP, and NPQ/4 in order to assess the efficiency and status of photosynthesis (F. He et al., 2019).

*Methods S10* *Leaf pre-processing for SEM photography*

To prevent the wax on the leaf surface from being washed away by organic solvents, we use liquid nitrogen to rapidly fix the plant leaves. The specific method refers to the previously published method (F. He et al., 2018). Leaves from two-month-old seedlings were detached into wire netting and immediately fixed in liquid nitrogen. Then, the fixed samples were immediately put into an -80 °C Ultra-low Freeze Dryer (Biosafer-18A, Jiangsu, China (Mainland)) and fully dried for 24 h. Finally, the dried samples were coated with a thin layer of gold using a sputter coater (Quorum SC7620, UK).

*Methods S11 Observation of leaf longitudinal section staining*

Mature leaves from the same parts of 6-week-old wild-type and transgenic plants were selected, and the leaves were sandwiched in carrot squares of about 0.8 cm and sectioned with a sliding freezer sectioning machine; the cuticle was stained with toluidine blue to create a clear contrast because toluidine blue can color acids; the stained sections were observed and photographed using an optical microscope (BX53; OLYMPUS) to ensure that the image data collected could accurately reflect the characteristics of the cuticle of plant leaves.

*Methods S12* *Leaf pre-processing for GC-MS*

As previously described, the leaf surface wax was extracted from poplar leaves and analyzed by GC-MS (Grünhofer *et al.*, 2022). We analyzed the main components of the waxes and their precursors soluble in lipophilic solvents, including three groups of compounds: fatty acids, alcohols, and alkanes. The quantitative analysis of specific compounds was accomplished by querying the relevant data in the PUBChem database and combining the automatic integration of peak areas with the internal standard method (Song *et al.*, 2022).

*Methods S13* *Transcriptome analysis*

RNA was collected from leaves of normally growing one-month-old OX-*PtrC2H2.2-6* and wild-type 84K and analyzed them by transcriptome sequencing. RNA sequencing data were acquired from Majorbio Biopharm (Shanghai, China). Clean reads were mapped to the P. trichocarpa genome (https://phytozome-next.jgi.doe.gov/info/Ptrichocarpa_v4_1). EdgeR was used to calculate DEGs. KEGG analyses were performed using the Meggie Online Cloud Platform (https://www.majorbio.com/web/www/index). RNA-Seq data can be found with accession number PRJNA1180278 in the Sequence Read Archive (https://www.ncbi.nlm.nih.gov/sra/).

*Methods S14 Expression patterns of PtrC2H2.2 and its reciprocal proteins following drought stress*

The expression of the *PtrC2H2.2-6* and its predicted interacting proteins after drought stress treatments was obtained from the *P. trichocarpa* Database (https://ngdc.cncb.ac.cn/ppgr/). For the drought treatment: RNA-seq and analysis of mature leaves of the same parts of poplar after untreated, short-term, and long-term drought treatments. Specific expression profiles are presented in Table S6. The visualization of the *PtrC2H2.2-6* and its predicted interacting proteins heat map was accomplished employing TBtools v2.0 (C. Chen et al., 2020).

*Methods S15* *Dual luciferase reporter assay*

A *PtrCYP86A7*pro fragment containing the CTCTCTCACT motif and a *PtrCYP86A8*pro fragment containing the CACCCTCACT motif were inserted into the pGreenll 0800-LUC vectors to generate the reporter construct (pGreenll 0800-LUC-*PtrCYP86A7*pro and pGreenll 0800-LUC-*PtrCYP86A8*pro). 35Spro:*PtrC2H2.2-6* effector was generated by recombinantly inserting the *PtrC2H2.2-6* gene into the pGreenll 62-SK vector. Transformation and dual-luciferase (LUC) activity determination were conducted as described elsewhere (X. Zhang et al., 2018). In brief, the recombinant plasmids were introduced into A. tumefaciens GV3101 (pSoup-p19) that were then cultured until an optical density at 600 nm of 1.2. The reporter and effector were combined in equal volumes, maintained at 20°C without shaking for 3 h, and instantly transfected into *Nicotiana benthamiana* leaves, and the leaves were harvested after 1 day of darkness and one day of light. Leaf blades were coated with uniformly equal amounts of luciferin potassium salt solution left and right, darkened for 5 min, and the fluorescence intensity was photographed with the GelView 6000Plus (BLT, China). A dual-luciferase reporter assay system (Promega, Madison, WI) was used to measure the levels of LUC and Renilla luciferase (REN) activity. REN is a luciferase enzyme derived from the sea kidney (Renilla reniformis, a marine mollusk). It is often used as a reporter gene in biological research, especially in dual luciferase reporter gene detection systems. The ratio of LUC to REN was used to express the transactivation. Each experiment was conducted with a minimum of 9 duplicates, consisting of 3 biological replicates and 3 technical replicates.

To investigate the effect of *PtrPPK1* on the transcriptional activity of *PtrC2H2.2-6*, we performed the following experiments: Combination 1 (35S:*PtrPPK1* + 35Spro:*PtrC2H2.2-6* + effector reporter construct) and Combination 2 (35S: + 35Spro:*PtrC2H2.2-6* + effector reporter construct) were co-transformed into tobacco leaves. co-transformed tobacco leaves. Subsequently, the fluorescence intensity of the leaves was measured in the same way.

*Methods S16* *Bimolecular fluorescence complementation assay*

For bimolecular fluorescence complementation detection, the CDSs of both *PtrC2H2.2-6* and *PtrPPK1* were recombined with the C- and N-terminal regions of YFP (H. Wang et al., 2021). After co-transfection of the bacterial solution with tobacco (refer to Methods S14 for the detailed procedure), we utilized a confocal microscope (FV3000, Olympus) to detect fluorescent signals in the samples. The following lines of the argon ion laser were used: 488 nm for YFP and 488 nm for chlorophyll. Fluorescence was detected at 495-530 nm for YFP and 650-680 nm for chlorophyll.

*Methods S17* *Phosphorylation experiments*

Using Agrobacterium-mediated transient transformation, the same concentrations of 35S:*PtrC2H2.2-6* and 35S:*PtrPPK1* or 35S empty vector bacteriophage were transferred into tobacco (Nicotiana benthamiana) leaves of the same condition (Zhang et al., 2024). After tobacco co-transformation, leaves were harvested after one day of darkness and one day of light, and a treatment of 50 μM MG132 (to prevent protein degradation) was applied to the leaves after 12 h of light leaf exposure. Total poplar protein was extracted using RIPA containing 1 mM PMSF, and protein loading buffer (6x) was added for subsequent up-sampling. Samples were electrophoresed using 10% SDS-PAGE containing either no additives or 50 μM Phos-tag (ApexBio) and 40 μM MnCl₂ and then transferred to PVDF membranes for immunoblot analysis.

**References**

**Chen C, Chen H, Zhang Y, Thomas HR, Frank MH, He Y, Xia R. 2020.** TBtools: An Integrative Toolkit Developed for Interactive Analyses of Big Biological Data. *Molecular Plant* **13**(8): 1194-1202.

**Ding S, Zhang B, Qin F. 2015.** Arabidopsis RZFP34/CHYR1, a Ubiquitin E3 Ligase, Regulates Stomatal Movement and Drought Tolerance via SnRK2.6-Mediated Phosphorylation. *The Plant Cell* **27**(11): 3228-3244.

**Duan M, Zhang R, Zhu F, Zhang Z, Gou L, Wen J, Dong J, Wang T. 2017.** A Lipid-Anchored NAC Transcription Factor Is Translocated into the Nucleus and Activates Glyoxalase I Expression during Drought Stress. *The Plant Cell* **29**(7): 1748-1772.

**Grünhofer P, Herzig L, Sent S, Zeisler‐Diehl VV, Schreiber L. 2022.** Increased cuticular wax deposition does not change residual foliar transpiration. Plant, Cell & *Environment* **45**(4): 1157-1171.

**He** **F, Wang H-L, Li H-G, Su Y, Li S, Yang Y, Feng C-H, Yin W, Xia X. 2018.** PeCHYR1, a ubiquitin E3 ligase from Populus euphratica, enhances drought tolerance via ABA-induced stomatal closure by ROS production in Populus. *Plant Biotechnology Journal* **16**(8): 1514-1528.

**He F, Li H-G, Wang J-J, Su Y, Wang H-L, Feng C-H, Yang Y, Niu M-X, Liu C, Yin W, et al. 2019.** PeSTZ1, a C2H2-type zinc finger transcription factor from Populus euphratica, enhances freezing tolerance through modulation of ROS scavenging by directly regulating Pe2. *Plant Biotechnology Journal* **17**(11): 2169-2183.

**He F, Niu M-X, Feng C-H, Li H-G, Su Y, Su W-L, Pang H, Yang Y, Yu X, Wang H-L, et al. 2020.** PeSTZ1 confers salt stress tolerance by scavenging the accumulation of ROS through regulating the expression of PeZAT12 and PeAPX2 in Populus. *Tree Physiology* **40**(9): 1292-1311.

**He F, Niu M-X, Wang T, Li J-L, Shi Y-J, Zhao J-J, Li H, Xiang X, Yang P, Wei S-Y. 2024.** The ubiquitin E3 ligase RZFP1 affects drought tolerance in poplar by mediating the degradation of the protein phosphatase PP2C-9. *Plant physiology* **196**(4): 2936-2955.

**Li J-L, Li H, Zhao J-J, Yang P, Xiang X, Wei S-Y, Wang T, Shi Y-J, Huang J, He F. 2024.** Genome-wide identification and characterization of the RZFP gene family and analysis of its expression pattern under stress in Populus trichocarpa. *International Journal of Biological Macromolecules* **255**: 128108.

**Liu J, Zhang C, Wei C, Liu X, Wang M, Yu F, Xie Q, Tu J. 2016.** The RING finger ubiquitin E3 ligase OsHTAS enhances heat tolerance by promoting H_2_O_2_-induced stomatal closure in rice. *Plant physiology* **170**(1): 429-443.

**Meng S, Cao Y, Li H, Bian Z, Wang D, Lian C, Yin W, Xia X. 2019.** PeSHN1 regulates water-use efficiency and drought tolerance by modulating wax biosynthesis in poplar. *Tree Physiology* **39**(8): 1371-1386.

**Shi H, Ye T, Chen F, Cheng Z, Wang Y, Yang P, Zhang Y, Chan Z. 2013.** Manipulation of arginase expression modulates abiotic stress tolerance in Arabidopsis: effect on arginine metabolism and ROS accumulation. *Journal of Experimental Botany* **64**(5): 1367-1379.

**Shi H, Ye T, Zhu J-K, Chan Z. 2014.** Constitutive production of nitric oxide leads to enhanced drought stress resistance and extensive transcriptional reprogramming in Arabidopsis. *Journal of Experimental Botany* **65**(15): 4119-4131.

**Song Q, Kong L, Yang X, Jiao B, Hu J, Zhang Z, Xu C, Luo K. 2022.** PtoMYB142, a poplar R2R3-MYB transcription factor, contributes to drought tolerance by regulating wax biosynthesis. *Tree Physiology* **42**(10): 2133-2147.

**Tong S, Chen N, Wang D, Ai F, Liu B, Ren L, Chen Y, Zhang J, Lou S, Liu H. 2021.** The U‐box E3 ubiquitin ligase PalPUB79 positively regulates ABA‐dependent drought tolerance via ubiquitination of PalWRKY77 in Populus. *Plant Biotechnology Journal* **19**(12): 2561-2575.

**Wang H-L, Zhang Y, Wang T, Yang Q, Yang Y, Li Z, Li B, Wen X, Li W, Yin W. 2021.** An alternative splicing variant of PtRD26 delays leaf senescence by regulating multiple NAC transcription factors in Populus. *The Plant Cell* **33**(5): 1594-1614.

**Zhang X, Ji Y, Xue C, Ma H, Xi Y, Huang P, Wang H, An F, Li B, Wang Y. 2018.** Integrated regulation of apical hook development by transcriptional coupling of EIN3/EIL1 and PIFs in Arabidopsis. *The Plant Cell* **30**(9): 1971-1988.

**Zhang N, Wei C-Q, Xu D-J, Deng Z-P, Zhao Y-C, Ai L-F, Sun Y, Wang Z-Y, Zhang S-W. 2024.** Photoregulatory protein kinases fine-tune plant photomorphogenesis by directing a bifunctional phospho-code on HY5 in Arabidopsis. Developmental Cell **59**(13): 1737-1749. e1737.
